# Mechanisms of PI(4,5)P2 Enrichment in HIV-1 Viral Membranes

**DOI:** 10.1016/j.jmb.2020.07.018

**Published:** 2020-07-31

**Authors:** Yi Wen, Gerald W. Feigenson, Volker M. Vogt, Robert A. Dick

**Affiliations:** Department of Molecular Biology & Genetics, Cornell University, Ithaca, NY 14853, USA

**Keywords:** human immunodeficiency virus, matrix protein, myristoylation, giant unilamellar vesicle, large unilamellar vesicle

## Abstract

Phosphatidylinositol 4,5-bisphosphate (PIP2) is critical for HIV-1 virus assembly. The viral membrane is enriched in PIP2, suggesting that the virus assembles at PIP2-rich microdomains. We showed previously that in model membranes PIP2 can form nanoscopic clusters bridged by multivalent cations. Here, using purified proteins we quantitated the binding of HIV-1 Gag-related proteins to giant unilamellar vesicles containing either clustered or free PIP2. Myristoylated MA strongly preferred binding to clustered PIP2. By contrast, unmyristoylated HIV-1 MA, RSV MA, and a PH domain all preferred to interact with free PIP2. We also found that HIV-1 Gag multimerization promotes PIP2 clustering. Truncated Gag proteins comprising the MA, CA, and SP domains (MACASP) or the MA and CA domains (MACA) induced self-quenching of acyl chain-labeled fluorescent PIP2 in liposomes, implying clustering. However, HIV-1 MA itself did not induce PIP2 clustering. A CA inter-hexamer dimer interface mutation led to a loss of induced PIP2 clustering in MACA, indicating the importance of protein multimerization. Cryo-electron tomography of liposomes with bound MACA showed an amorphous protein layer on the membrane surface. Thus, it appears that while protein–protein interactions are required for PIP2 clustering, formation of a regular lattice is not. Protein-induced PIP2 clustering and multivalent cation-induced PIP2 clustering are additive. Taken together, these results provide the first evidence that HIV-1 Gag can selectively target pre-existing PIP2-enriched domains of the plasma membrane for viral assembly, and that Gag multimerization can further enrich PIP2 at assembly sites. These effects could explain the observed PIP2 enrichment in HIV-1.

## Introduction

Assembly of retroviruses, such as human immunodeficiency type 1 (HIV-1), is driven by the structural polyprotein Gag. Approximately 2500 copies of Gag assemble at the plasma membrane (PM) to form an immature virion, with the N-terminal MA domain associating with the inner leaflet of the PM, the central CA domain forming a hexameric lattice arranged as an incomplete sphere, and the C-terminal end of Gag bound to viral genomic RNA [4,5]. During or immediately after budding, the virus undergoes major rearrangements triggered by cleavage of Gag by the viral protease resulting in the formation of a mature virion. For HIV-1, the PM phospholipid PI(4,5)P2 (referred to throughout as PIP2) is enriched in the viral membrane approximately three-fold in comparison with the PM [6]. A recent study estimated that virus particles contain three times as many PIP2 molecules as total Gag molecules [7]. PIP2 depletion in cells reduces both HIV-1 Gag PM localization and virus release, redirecting Gag to intracellular membranes [8,9]. Super-resolution live cell imaging also suggests that for HIV-1 assembly site formation is highly dependent on PIP2 and that HIV-1, Gag specifically restricts PIP2 mobility in living cells [10,11]. Consistent with *in vivo* work, PIP2 strongly enhances the binding of Gag and its derivative proteins to liposomes *in vitro* [9,12–14]. Thus, we and others have speculated that in assembly, HIV-1 Gag specifically targets membrane microdomains that are enriched in PIP2 [15].

The MA domain of HIV-1 directs Gag to the negatively charged inner leaflet of the PM through a bipartite signal, an N-terminal 14-carbon myristate modification and a nearby patch of basic residues [16]. The insertion of myristate into membranes, providing ~8 kcal/mol in free energy, is essential, since mutation of the glycine at position 2 blocks myristoylation [17] and leads to loss of PM budding and reduction in membrane binding *in vitro* [18–20]. According to surface plasmon resonance and liposome flotation assays, MA myristoylation increases the membrane affinity by a factor of 10 [18]. While this fatty acyl modification is necessary, it is not sufficient for stable membrane binding. The patch of basic residues on the globular head of MA, including the highly basic region (HBR) spanning residues 15–31, also is critical for efficient membrane binding [21]. These amino acids, exposed on the surface of the protein [21,22], interact with negatively charged lipids in the inner leaflet, phosphatidylserine (PS) and PIP2 [9,13,23,24]. The relative contributions of electrostatic interactions and the hydrophobic interactions have been difficult to decipher [25–27].

HIV-1 MA is reported to contain a specific PIP2 binding pocket [28] or binding surface [29], which prominently includes residues K30 and K32 (or K29 and K31). Myristoylated Gag with mutations in these crucial amino acids displays an almost 3-fold decrease in binding to PIP2-containing liposomes [9,14]. Interestingly, a Gag mutant with all HBR lysines and arginines switched with each other (HBR/RK switch) exhibits almost no binding to PIP2-containing liposomes. This result indicates that not only the overall charge but also the specific amino acid sequence within HBR is critical for Gag–PIP2 interaction [13,30]. In addition, the PIP2-binding site overlaps an RNA-interaction site in MA [31]. RNA bound to the MA blocks interactions with PS-containing membranes, but not with PIP2-containing membranes, suggesting that RNA might serve as a regulator to ensure that Gag targets PIP2-rich sites at the PM [32,33]. Overexpression of polyphosphoinositide 5-phosphatase IV (5ptaseIV), which depletes PIP2 at the PM, abrogates HIV-1 virus particle release and redirects Gag to intracellular compartments [8]. According to several lipidomic studies, PIP2 enrichment in HIV-1 viral membranes is MA-dependent [6,34,35]. Altogether, these results demonstrate that the specific interaction between HIV-1 MA and PIP2 is essential for correct PM targeting and virus assembly.

In addition to directing HIV-1 Gag to the PM, the MA domain also is involved in incorporation of envelope (Env) proteins into virus particles [36,37]. While the mechanism of Env localization to assembly sites remains unclear, many studies provide evidence of a direct interaction of HIV-1 MA and the cytoplasmic tail (CT) of Env [38–50]. Assembly-competent MA mutants that fail to incorporate Env can be rescued by large C-terminal truncations of the CT of Env or by compensatory mutations in MA [48]. Similarly, Env mutants with small deletions in the CT, leading to Env-deficient particles, can be rescued by mutations in MA [48]. Multimerized Gag reduces Env mobility at assembly sites [51], consistent with the idea that the MA domain interacts with, and retains, Env in the nascent virus.

Both the CA and the short adjoining SP1 domains are critical for HIV-1 Gag multimerization [52,53]. Like all retroviral CA proteins, HIV-1 CA is composed of two distinct folded sub-domains, often called the CA_NTD_ and CA_CTD_, respectively. Assembly-defective mutations in CA are clustered in three regions: a surface composed of helices H4 to H6 in CA_NTD_, the CA dimer interface in CA_CTD_, and the loop preceding H8 at the base of CA_CTD_ [54,55]. The dimer interface is critically important for particle assembly, maturation, and infectivity [53,56–59]. The short conserved region SP1, just C-terminal to CA, together with seven residues upstream, fold into a six-helix bundle (6HB), which helps form and stabilize the immature Gag hexamer [60–63]. The 6HB is required not only for proper assembly, but also for Gag-PM association [64,65], apparently due to the decreased membrane affinity resulting from a defect in multimerization [64].

PIP2 has been proposed to exist as spatially separated pools [66]. Recently, we showed by self-quenching and Förster resonance energy transfer (FRET) techniques that PIP2 forms clusters in model membranes that resemble the inner leaflet of the PM [67]. This clustering is unique to phosphatidyl inositol species. Clustering occurs even at the extremely low PIP2 concentration of 0.05% of total lipid, is dependent on the presence of multivalent cations, and is influenced by the surrounding lipid environment. For example, clustering occurs under physiological conditions of pH 7.2, 100 mM KCl, 0.5 mM Mg^2+^, and 2 mol% PIP2 in an inner leaflet model membrane. These results suggest that in living cells, PIP2 most likely exists both in free and in clustered forms in the PM. Other than multivalent cations, numerous cellular PIP2 binding proteins are also proposed to modulate PIP2 distribution and induce PIP2 cluster formation, either by sequestration electrostatically, such as MARCKS [68], or by protein multimerization, like the pleckstrin homology (PH) domain of dynamin [69]. However, the contribution of electrostatic interaction together with protein multimerization has not been carefully examined in the case of PIP2 enrichment at HIV-1 Gag assembly sites.

In the present work, we used purified HIV-1 Gag derivatives and model membranes to study the interplay of the PIP2–PIP2, Gag–PIP2, and Gag–Gag interactions that may take place during HIV-1 assembly (A diagram of the key experimental setups is shown in Figure S1). Giant unilamellar vesicles (GUVs) were used for protein fluorescence binding assays, and large unilamellar vesicles (LUVs) were used for cuvette-based fluorescence quenching assays. First, we found that HIV-1 Gag shows preference for clustered PIP2 over free PIP2, in contrast to other PIP2 binding proteins. We then showed that Gag multimerization on model membranes induces PIP2 clustering. Taken together, these results provide a mechanistic underpinning of PIP2 enrichment in HIV-1 viral membranes.

## Results

### PIP2 can form multivalent cation-dependent clusters in the absence of proteins

**Figure 1.**
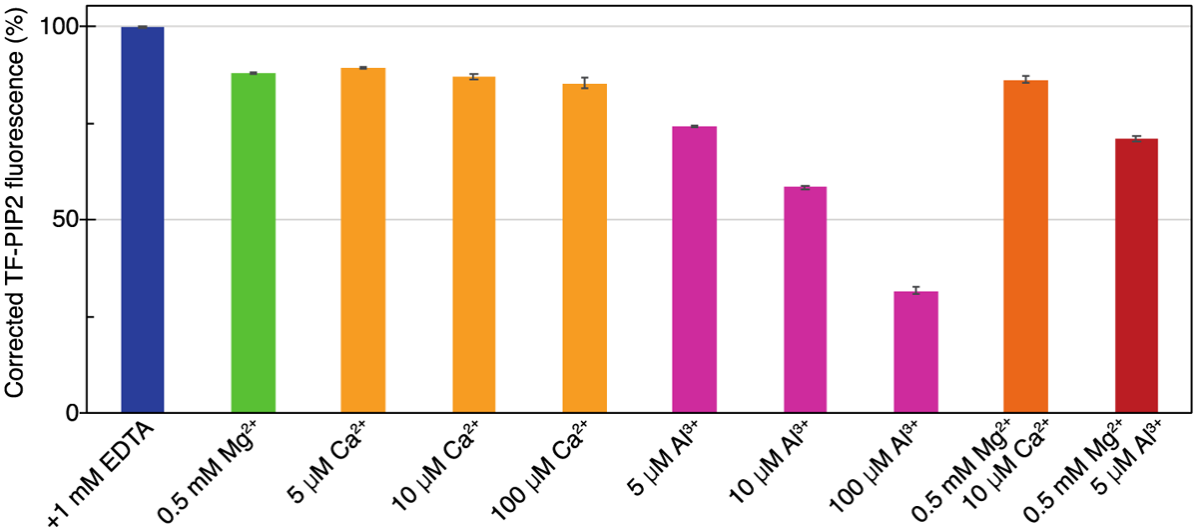
Physiological levels of PIP2 form clusters bridged by multivalent cations in inner leaflet model membranes. LUVs were composed of POPE/POPS/Chol/PIP2 (32/30/36/2). The 2 mol% total PIP2 was a mixture of Brain-PIP2 and TF-PIP2 at a 7/3 ratio. LUVs were prepared in each buffer independently at a final concentration of 500 μM lipid. All buffers were based on 100 mM KCl, 20 mM HEPES (pH 7.2), with additional EDTA or multivalent cations as shown. All buffers were prepared with high-purity chemicals and stored in Teflon bottles. TF-PIP2 fluorescence of LUVs in EDTA was set to 100%, while TF-PIP2 fluorescence of LUVs in other buffer conditions was converted to a percentage relative to the maximum fluorescence in EDTA. Note that TF-PIP2 fluorescence quenching was from both inner and outer leaflets of LUVs, as both leaflets were exposed to buffers. Assays were performed at room temperature at least three times with error bars of standard deviations from the means. Slits of Ex/Em 485/515 were 2.5/2.5 nm in these and all similar experiments.

PIP2 is hypothesized to exist as “spatially separated pools” at the inner leaflet of the PM [70]. Our previous study showed that physiological levels of PIP2 can form clusters in the presence of multivalent cations, as shown both by self-quenching and by FRET [67]. We first detected this clustering using a fluorescence self-quenching assay. We prepared 100 nm LUVs with an inner leaflet lipid composition POPE/POPS/Chol/PIP2 (32/30/36/2), and tested fluorescence quenching at different ratios of natural brain PIP2 and fluorescently labeled TopFluor PIP2 (TF-PIP2), always at final total 2 mol % PIP2 (Figure S2A). All of these fluorescence quenching assays were carried out at a ratio of brain-PIP2/TF-PIP2 = 7/3, which gives modest quenching in the presence of multivalent cations that are present on both leaflets of LUVs. Buffers were prepared to mimic the ions and pH of the cell: 100 mM KCl, 20 mM HEPES, (pH 7.2), either with addition of 1 mM EDTA or of multivalent cations (Figure 1). The fluorescence from TF-PIP2 in each buffer was converted into a percentage relative to the maximum fluorescence from TF-PIP2 in EDTA, in which PIP2 is known to be free (unclustered) [67]. Reduction of TF-PIP2 fluorescence due to self-quenching indicates PIP2 clustering. The buffer with 0.5 mM Mg^2+^ and 10 μM Ca^2+^, modeling a physiological transient calcium influx in cells, gave moderate PIP2 clustering, while the buffer with 0.5 mM Mg^2+^ and 5 μM Al^3+^ gave stronger PIP2 clustering, as reported previously [67]. Al^3+^ was used, as it was previously shown to cause the greatest amount of PIP2 clustering as measured by quenching. Under all tested buffer conditions, GUVs labeled with TF-PIP2 look uniform, implying that these PIP2-cation clusters are nanoscopic, below optical resolution (Figure S2B). In summary, a physiological level of PIP2 can form PIP2-cation clusters on membranes in the absence of protein binding. PIP2 lateral organization, i.e. extent of clustering, can be modulated experimentally by controlling multivalent cation type and concentration.

### Myristoylated HIV-1 MA preferentially binds to clustered PIP2 over free PIP2

**Figure 2.**
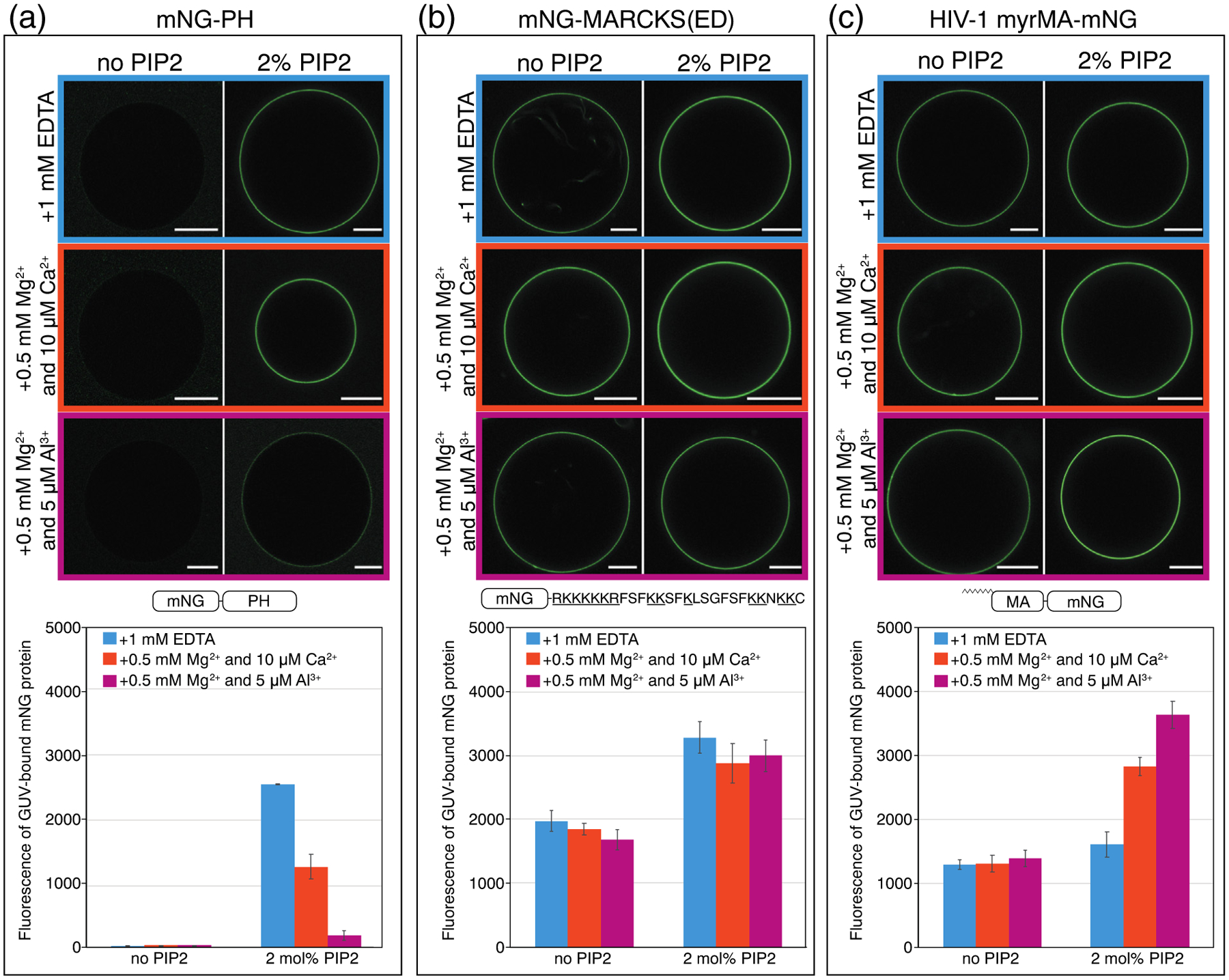
Myristoylated HIV-1 MA (myrMA) prefers binding to clustered PIP2, whereas the pleckstrin homology domain from phospholipase C (PH) favors binding to free PIP2. The myristoylated alanine-rich C kinase substrate effector domain (MARCKS(ED)) shows no preference. GUVs were prepared with no PIP2 [POPE/POPS/Chol (34/30/36)] or with 2 mol% PIP2 [POPE/POPS/Chol/brain-PIP2 (32/30/36/2)]. All GUVs were labeled with the membrane dye 18:1 DiI at a dye/lipid ratio of 1/2500. Both types of GUVs were harvested into one of three buffer conditions 5 h prior to protein binding. All three buffers were based on 100 mM KCl, 20 mM HEPES (pH 7.2), with either EDTA or multivalent cations. For GUVs incubated with 1 mM EDTA (top row), PIP2 is free; for GUVs incubated with 0.5 mM Mg^2+^ and 10 μM Ca^2+^ (middle row), PIP2 is modestly clustered; for GUVs incubated with 0.5 mM Mg^2+^ and 5 μM Al^3+^ (bottom row), PIP2 is strongly clustered. All proteins used in GUV assays were fused with mNG, with constructs shown in (a) mNG-PH, (b) mNG-MARCKS(ED), and(c) HIV-1 myrMA-mNG. Each mNG-labeled protein was added to the outside of GUVs at a final concentration of 1 μM. GUVs with protein bound were subject to confocal imaging under the same setting. Each GUV was examined at 488 nm for mNG fluorescence and at 561 nm for DiI fluorescence for locating GUVs. Fluorescence intensity above background (measured outside the GUV) at 488 nm (mNG) was analyzed by line scans in ImageJ across the perimeter of each GUV. The values were averaged and plotted with error bars representing the standard deviation. For each protein, a higher mNG fluorescence intensity indicates a higher membrane binding affinity. For GUVs with no PIP2, at least 15 GUVs were analyzed, and for GUVs with 2 mol% PIP2, at least 30 GUVs were analyzed, under each buffer condition. GUVs for each condition were prepared at least three times independently.

We asked how HIV-1 MA and other PIP2 binding proteins respond to the lateral distribution of PIP2 in GUVs. The proteins were expressed and purified from *Escherichia coli* as fusions with monomeric Neon Green (mNG) [71]. GUVs with model PM inner leaflet lipid composition were prepared with or without 2% unlabeled natural (brain-derived) PIP2. Three buffer conditions were used to give different PIP2 lateral distributions [67]: 1.0 mM EDTA (free PIP2), 0.5 mM Mg^2+^ and 10 μM Ca^2+^ (moderately clustered PIP2), and 0.5 mM Mg^2+^ and 5 μM Al^3+^ (strongly clustered PIP2). All protein-GUV binding assays were carried out at 1 μM final protein concentration (Figure 2). All proteins tested have measurable membrane binding at this protein concentration. The binding is below saturation at 100 mM KCl. The fluorescence intensity of bound protein on membranes is optimal for visualization under the conditions used. In the absence of PIP2, the membrane binding of each protein was very similar under these three buffer conditions (Figure 2), suggesting that the absence or presence of multivalent cations does not influence protein interaction in the bulk lipid environment (PE, PS, and Chol).

We found that some proteins can sense PIP2 lateral distribution and others cannot. The phospholipase Cδ1 PH domain (mNG-PH) is the best characterized, specific PIP2 binding protein, having a structured binding pocket for the PIP2 headgroup [72,73]. As expected, mNG-PH showed almost no binding to membranes without PIP2. This protein bound most extensively to GUVs with free PIP2, in the presence of EDTA, with approximately 2-fold reduction in binding to moderately clustered PIP2, and almost 10-fold reduction to strongly clustered PIP2 (Figure 2(a)). Thus, it appears that cation-bridged PIP2 clusters hinder specific PH-PIP2 interactions. This result agrees with a previous study showing that Ca^2+^ can confine the PIP2 headgroup tilt angle and inhibit PH recognition and binding, as evidenced both by liposome experiments and molecular dynamics simulations [74]. In contrast to PH-mNG, the well-characterized MARCKS effector domain (amino acids 151–175) peptide, termed MARCKS(ED) [75], fused to mNG was not sensitive to PIP2 clustering, with apparently equal membrane affinity for all three PIP2 clustering conditions (Figure 2(b)), even though MARCKS has been reported to sequester approximately three PIP2 headgroups [76,77]. Thus, it is possible that the preference for PIP2 lateral organization by each protein is different than its ability to sequester PIP2 laterally.

**Figure 3.**
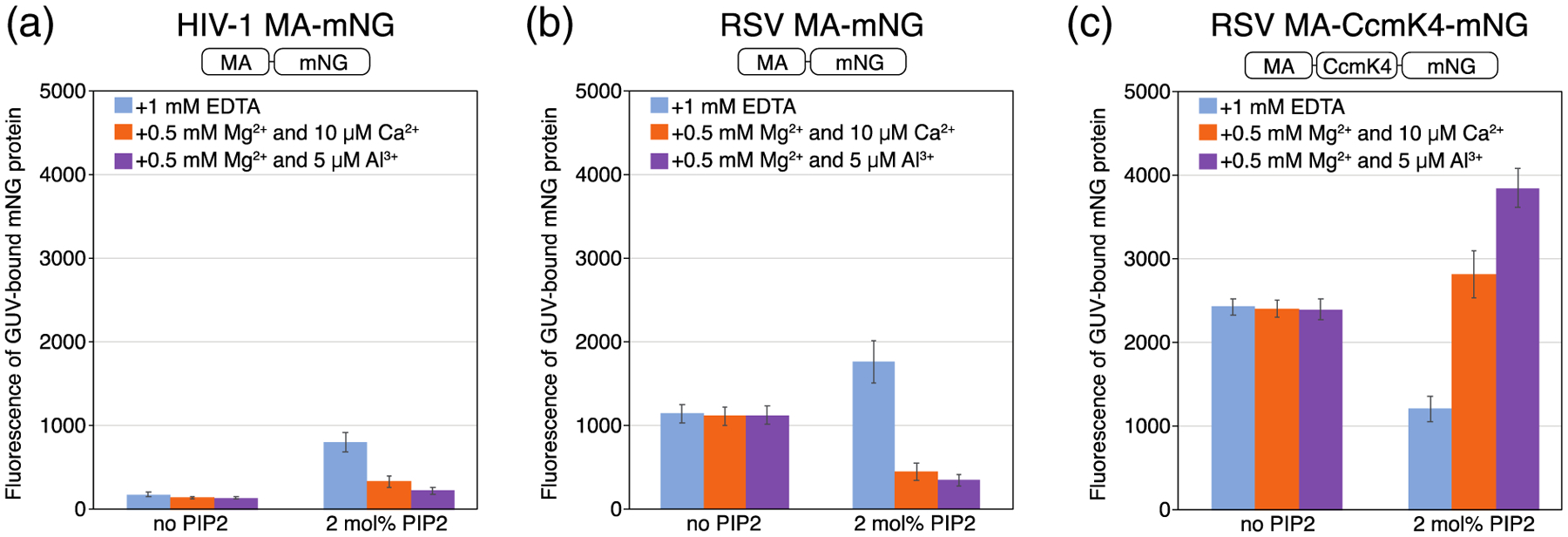
Sensitivity of non-myristoylated and multimerized protein to PIP2 clustering. Quantification of mNG-labeled protein bound to GUVs was carried out as described in Figure 3. (a) Non-myristoylated HIV-1 MA-mNG binding in the absence and presence of 2% PIP2 without (EDTA) or with multivalent cations. (b) Naturally non-myristoylated RSV MA-mNG binding as in (a). (c) Synthetically hexamerized RSV MA-CcmK4-mNG protein binding as in (a).

The membrane affinity of the naturally myristoylated HIV-1 MA protein (myrMA) was clearly sensitive to PIP2 lateral organization (Figure 2(c)). In GUVs with 2mol% PIP2, myristoylated HIV-1 MA had the highest affinity for membranes containing strongly clustered PIP2, with almost a 2-fold increase over membranes in EDTA (Figure 2(c)). We interpret these data to mean that HIV-1 myrMA prefers binding to clustered PIP2 over free PIP2. As expected, HIV-1 MA lacking myristoylation had much lower GUV binding at the same protein concentration, due to a loss of the binding energy from the hydrophobic myristate insertion into the membrane. Strikingly, however, HIV-1 MA without the N-terminal myristate exhibited the opposite behavior as myrMA, with highest affinity for membranes containing free PIP2, almost 4-fold higher than for strongly clustered PIP2 (Figure 3(a)). We also assayed MA from Rous sarcoma virus (RSV), which is not naturally myristoylated. Monomeric RSV MA-mNG behaved like unmyristoylated HIV-1 MA, with a 5-fold higher binding to free PIP2 than to clustered PIP2 (Figure 3(b)). However, a hexameric version of the same protein, RSV MA-CcmK4-mNG, behaved similarly to HIV-1 myrMA, exhibiting the highest affinity for membranes containing strongly clustered PIP2. This protein was created by fusing MA with the cyanobacterial carboxysome shell protein Ccmk4 (Figure 3(c)). RSV MA-CcmK4-mNG has an enhanced membrane binding, as reported previously both *in vitro* and *in vivo* [78].

**Figure 4.**
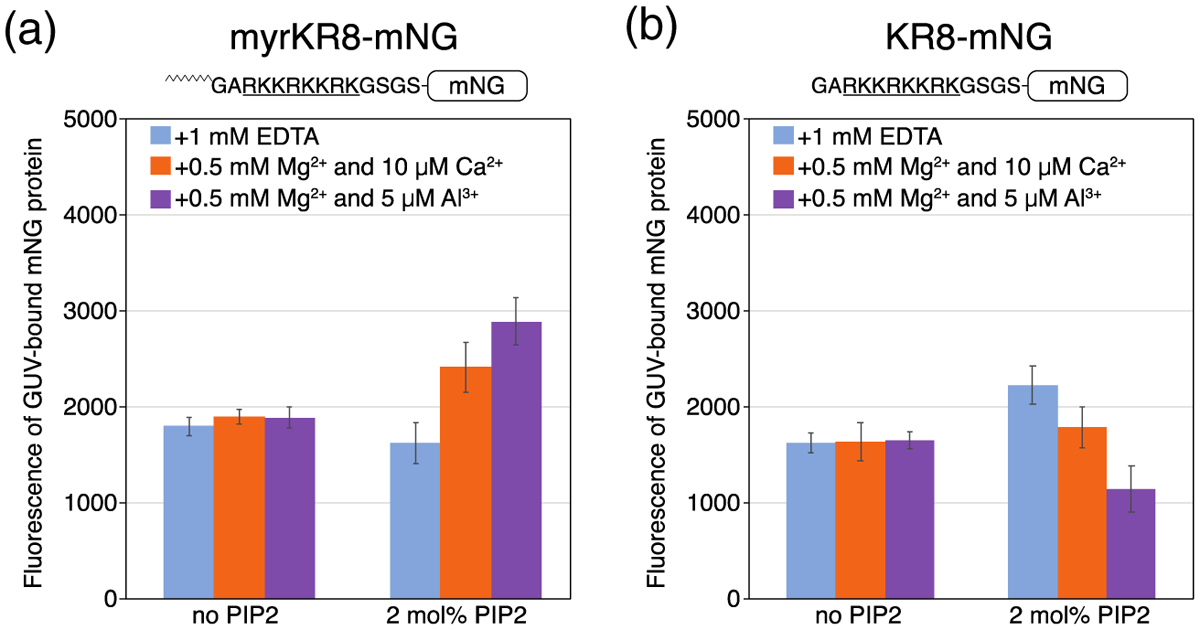
Myristoylation confers binding preference to clustered PIP2. Quantification of protein binding to GUVs with the same composition and buffer conditions was carried out as detailed in Figure 3. (a) Myristoylated KR8-mNG protein and (b) non-myristoylated KR8-mNG protein. The myristoylation signal at the N-terminus for the protein (amino acids GAR) is the same as in HIV MA.

To test independently whether this preference for PIP2 clusters is a consequence of myristoylation, we created a pair of electrostatic sensor proteins comprising mNG with eight consecutive lysine or arginine residues, with or without an N-terminal myristate, myrKR8-mNG and KR8-mNG, respectively. Similar to HIV-1 myrMA, myrKR8-mNG preferentially bound to clustered PIP2, showing approximately 2-fold more binding with strongly clustered PIP2 than with free PIP2 (Figure 4(a)). Similar to HIV-1 MA, KR8-mNG without the myristate modification preferred binding to free PIP2 (Figure 4(b)). The mechanism by which myristate modulates preference for PIP2 lateral organization in both HIV-1 MA and KR8-mNG is unclear. We interpret this result to support the hypothesis that proteins interacting most strongly with membranes, such as those with bipartite signals or that multimerize, can compete with multivalent cations for interaction with PIP2 clusters.

### Multimerization of HIV-1 Gag proteins induces PIP2 clustering in the absence of multivalent cations

**Figure 5.**
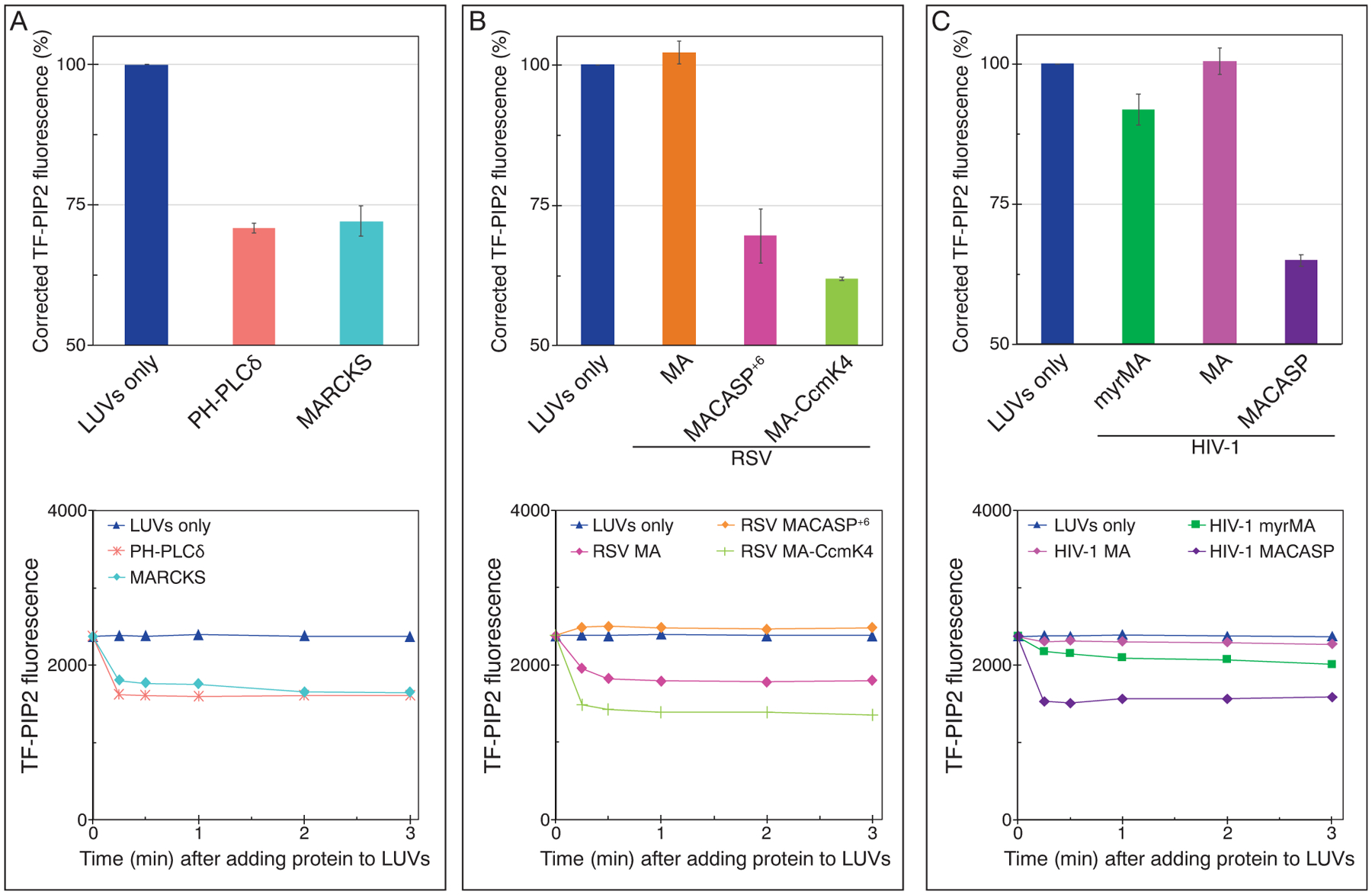
HIV-1 and RSV Gag-derivative proteins can induce PIP2 clustering, as measured by fluorescence quenching. LUVs with 2% PIP2 were prepared as in Figure 2 using 1 mM EDTA to eliminate all multivalent cation binding. Protein at a final concentration of 20 μM was added after LUV formation, and thus the inner leaflet of the liposomes was not exposed to protein. Top panels: TF-PIP2 fluorescence quenching by (a) control proteins, (b) RSV membrane binding proteins, and (c) HIV-1 membrane binding proteins. TF-PIP2 fluorescence of LUVs mixed with buffer was set to 100%, and the effect of added protein was recorded. Note that for protein-induced PIP2 clustering, the *Y*-axis is shown only from 50% to 100%, since TF-PIP2 fluorescence quenching occurred only from the outer leaflet where lipids were exposed to the proteins. Each bar in the graph represents the average TF-PIP2 fluorescence % from three time points 1, 2, and 3 min post-mixing. Each quenching assay was performed at least three times with error bars of standard deviations from the means. (a–c, bottom panels) Time course of quenching. For each assay, TF-PIP2 fluorescence was measured at 0.25, 0.5, 1, 2, and 3 min post-mixing.

Evidence suggests that in cells, membrane-bound HIV-1 Gag co-localizes with PIP2 clusters [10]. However, it remains unknown if such co-localization is due to Gag binding to pre-existing PIP2 clusters, or alternatively is due to Gag enriching this lipid at assembly sites because of Gag lattice formation. Here, we sought to test, using model membranes, if HIV-1 Gag proteins can induce PIP2 to form clusters independently of multivalent cations. We made fluorometric measurements of 100 nm LUVs with POPE/POPS/Chol/PIP2 (32/30/36/2) at a ratio of brain-PIP2/TF-PIP2 = 7/3. In order to eliminate multivalent cation-induced PIP2 clustering, 1 mM EDTA was included in all buffers. In this cuvette assay, the maximum TF-PIP2 fluorescence in 400 μM total lipids, in the absence of protein, was defined as 100% (Figure 5). First, we tested both the PH domain and the 25-residue MARCKS(ED) peptide, which contains an unstructured basic surface [79]. Addition of 20 μM of either PH or MARCKS peptide led to a 30% drop in TF-PIP2 fluorescence (Figure 5(a)). This value is highly significant considering the precision of such cuvette assays, and that quenching occurs only from the outer leaflet of LUVs where lipid head groups are exposed to added protein (Figure 1). The observation that the PH domain both prefers binding free PIP2 (Figure 3) and can induce PIP2 to cluster is discussed in detail in the discussion section below. In a concentration series, MARCKS peptide induced similar levels of PIP2 clustering at concentrations as low as 5 μM (Figure S3). The fluorescence decrease occurred rapidly, within 15 s after mixing proteins with LUVs, as shown by time course measurements.

These results are consistent with a previous study, which reports that the addition of 0.5 μM of either PH or MARCKS peptide can induce about 30% quenching in 100 nm LUVs composed of 100 μM DOPC and 1 mol % caproic acid (C6)- or palmitic acid (C16)-BODIPY-TMR-PIP2 [69]. MARCKS is reported to electrostatically sequester three PIP2 headgroups upon binding [75,76,80,81], consistent with the 30% loss of fluorescence after addition of MARCKS ED. Our system has several advantages over that used in the previous report. First, the model membranes used here reflect the PM inner leaflet lipid composition where PIP2 is found, and include a high percentage, 70%, of the total PIP2 as the natural brain-PIP2. Second, the buffers contain EDTA, ensuring that PIP2 is in its free form prior to protein binding.

Next, we sought to test whether RSV Gag-derived proteins, which like HIV-1 are known to interact with PIP2, can induce PIP2 clustering. There is no evidence of multimerization of RSV MA, and RSV MA is believed to be monomeric in solution. Addition of 20 μM RSV MA failed to induce any PIP2 quenching (Figure 5(b)). However, RSV MACASP^+6^, a truncated Gag extending from the MA domain through the first 6 residues of NC, thus including the sequence that forms a 6HB [82], resulted in a dramatic 30% self-quenching (Figure 5(b)). This protein was shown previously to form hexamer-like contacts on membranes [83]. The self-quenching induced by MACASP^+6^ was concentration-dependent (Figure S4) in the range of 0.4 to 40 μM, with near maximum quenching occurring at 10 μM, suggesting saturation of protein–membrane binding at higher concentrations. Thus, we chose to test all further viral proteins at 20μM, a concentration that should nearly maximize the effect of proteins on PIP2 reorganization. To further explore the effect of protein multimerization on PIP2 clustering, we tested the artificial hexameric chimeric protein RSV MA-CcmK4 [78,84,85]. Indeed, this protein led to an even stronger degree of TF-PIP2 self-quenching than RSV MACASP^+6^ (Figure 5(b)), perhaps because MA-Ccmk4 can pre-assemble into hexamers in solution while RSV MACASP^+6^ probably multimerizes efficiently only on membranes. Collectively, these results imply that RSV Gag multimerization is key to protein-induced PIP2 clustering.

To explore the effect of HIV-1 Gag proteins on PIP2 lateral re-organization, we first prepared both myristoylated and non-myristoylated versions of MA. Neither of these proteins caused fluorescence quenching of the TF-PIP2 (Figure 5(c)). By contrast, addition of HIV-1 (non-myristoylated) MACASP, a Gag derivative extending from MA to the beginning of NC, led to a 35% drop in fluorescence, indicating a pronounced PIP2 clustering effect (Figure 5(c)). HIV-1 MACASP, similar to RSV MACASP^+6^, has the capability of multimerizing on membranes. In summary, for both RSV and HIV-1, Gag proteins capable of binding to membranes and multimerizing there promote PIP2 clustering. Thus, we infer that the retroviral structural protein Gag can enrich PIP2 at assembly sites on the PM.

In the process of carrying out these experiments, we made the surprising discovery that a C-terminal 6His tag on MA has a profound effect that leads to artifactual results. Most previously published reports on the biochemical or biophysical behavior of purified HIV-1 MA have been based on 6His-tagged protein [86–88]. In comparing C-terminally tagged and untagged MA, we found that the stretch of His residues leads not only to higher protein–membrane binding, but also to significant PIP2 clustering (Figure S5). This artifact was confirmed by testing purified myristoylated HIV-1 MA, non-myristoylated HIV-1 MA, and RSV MA, all with or without a C-terminal 6His tag. Thus, as with many other proteins, drawing convincing conclusions requires addressing possible adventitious effects of artificial peptide tags. For this reason, all proteins presented in this study, unless specifically stated otherwise, were not 6His tagged.

### Mutations in the HIV-1 MA-PIP2 binding site block Gag-induced clustering

**Figure 6.**
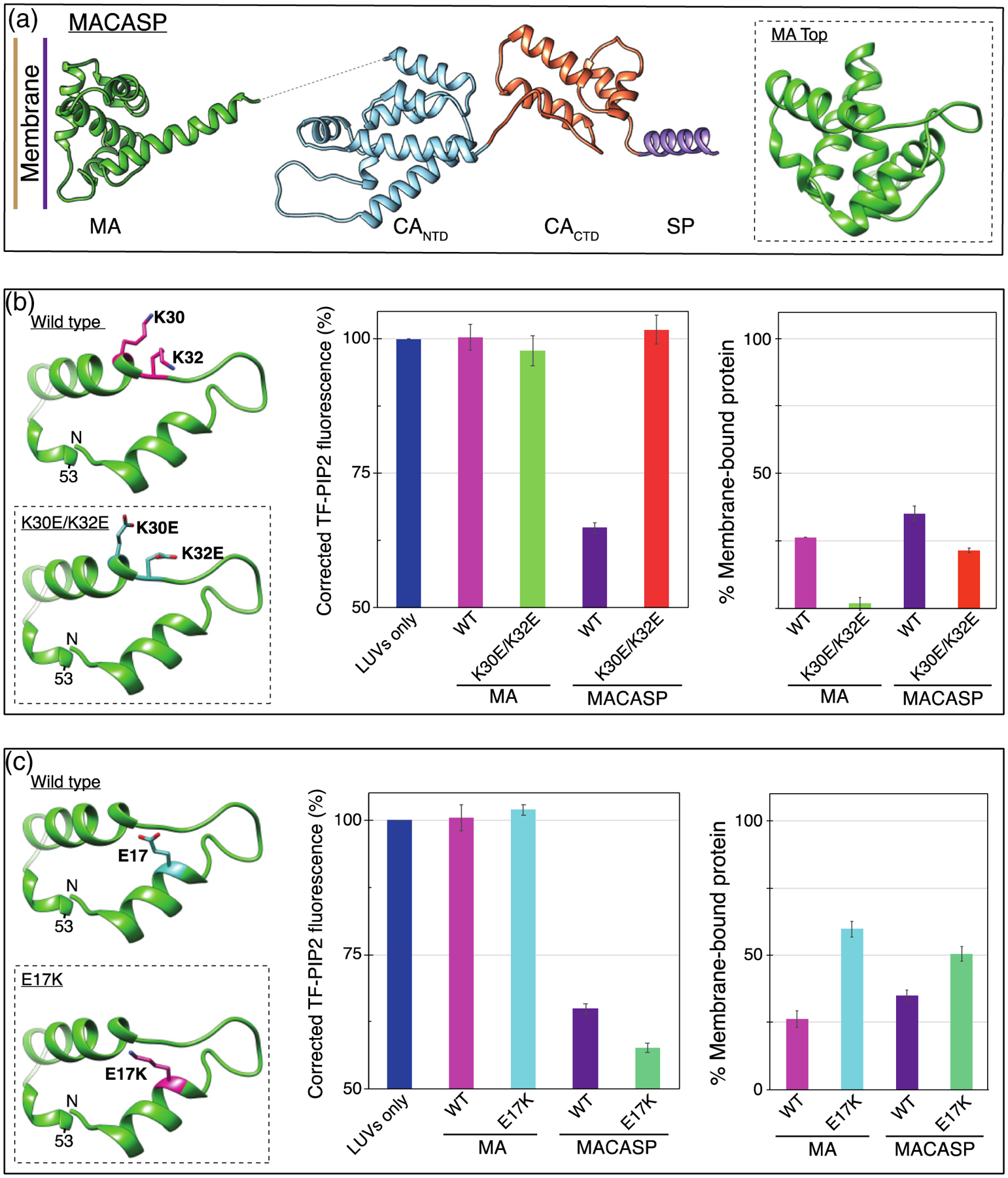
PIP2 clustering is dependent on known HIV MA PIP2-interacting amino acids. (a) Model of HIV-1 MACASP structure from PDB 1HIW (MA) [90] and 5L93 (CASP) [62]. Dotted line represents unstructured amino acids ~121–147. Inset is a top-down view of the membrane binding surface of MA. (b, left) MA membrane binding region, amino acids 7–53. PIP2-interacting amino acid K30 and K32 side-chains are shown in pink. Inset shows mutations K30E and K32E. (b, center) TF-PIP2 fluorescence in the absence and presence of wild-type (WT) or mutant HIV-1 MA or HIV-1 MACASP protein. (b, right) Membrane binding of each protein was determined by the pelleting assay. After incubating mixtures of 160μl LUVs and 40 μl proteins for 10 min, the protein-LUV mixture was ultracentrifuged at 75K for 15min at 4 °C. The supernatant was discarded and the pellet resuspended and subjected to SDS-PAGE and densitometry analyses. Each pelleting assay was performed at least three times with error bars of standard deviations from the means. (c, left) Amino acid E17 is shown in pink. Inset shows the membrane binding enhancement mutant E17K. (c, center) TF-PIP2 fluorescence in the absence and presence of WT or mutant HIV-1 MA or HIV-1 MACASP protein. (c, right) Membrane binding as described above.

We tried to elucidate the contribution of each Gag domain to the PIP2 clustering ability of HIV-1 Gag proteins. Amino acids K30 and K32 in MA have been reported to play an important role in Gag localization to the PM *in vivo* [26,50,89]. In cells, Gag with these residues mutated to acidic residues (K30E/K32E) (Figure 6) is reported to lack efficient particle assembly and release, and to be targeted to a multivesicular compartment instead of the PM. Furthermore, this mutant Gag shows reduced binding to PIP2-containing membranes *in vitro* [27]. We examined membrane binding of these dual KE mutants, both in the context of MA and of MACASP (Figure 6(a) and (b)). Consistent with previous reports, the mutant MA protein showed low membrane binding to PIP2-containing LUVs and, like the wild-type protein, caused no clustering of this lipid (Figure 6(b)). The same mutations in MACASP led to a 2-fold decrease in membrane binding, but the more dramatic effect was the complete loss of protein-induced PIP2 clustering (Figure 6(b)). We interpret this result to mean that direct MA-PIP2 interaction is critical for the induction of PIP2 clustering. Another well-known MA mutation, E17K (Figure 6(c)), is reported to lead to both enhanced membrane binding and more efficient virus release [50,91]. Consistent with published data, in our hands MA E17K increased membrane binding by about 2-fold (Figure 6(c)). This mutation in the context of MACASP caused a slight increase in PIP2 self-quenching, perhaps due to the observed increase in apparent membrane affinity.

**Figure 7.**
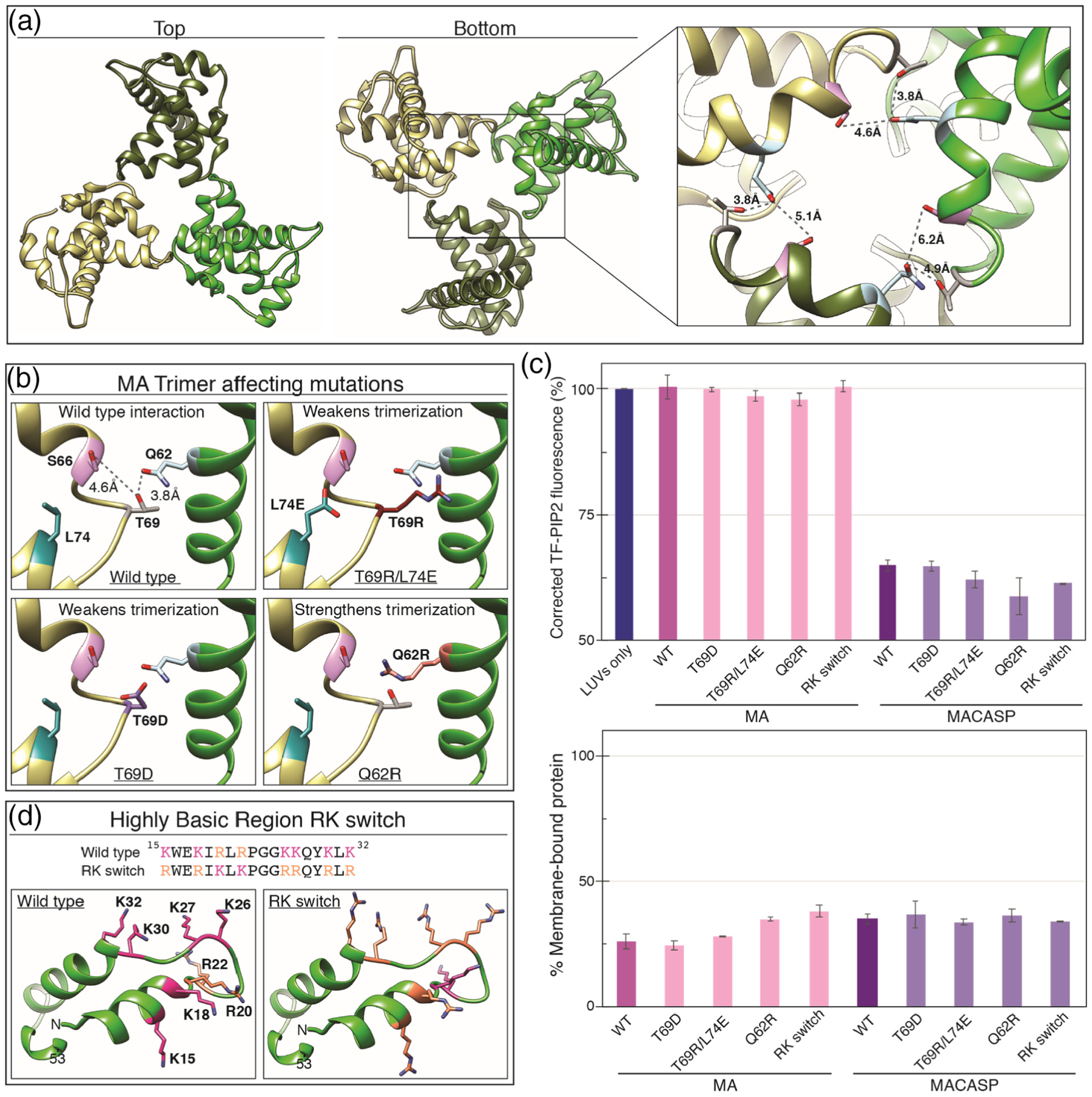
PIP2 clustering is not affected by mutations that weaken MA trimerization or alter charged residue type. (a) HIV-1 MA trimer (PDB 1HIW [90]) top and bottom views. Inset shows side-chains of known trimer interacting residues Q62 (blue), S66 (pink), T69 (grey) and Q62 (blue). (b) Mutations in MA that weaken or strengthen trimerization. (c) (Top) Effect of MA trimer mutations and RK switch mutant on PIP2 quenching in the context of MA and MACASP proteins. (Bottom) Percent of total protein associated with membranes. (d) Highly basic surface of the HIV-1 membrane binding domain. All basic amino acid side-chains are shown as pink (K) or orange (R). In the RK switch mutant, each K residue has been mutated to R and each R residue has been mutated to K.

We also examined the effects of several other known mutations in HIV-1 MA, both in the context of MA and of MACASP. One of the earliest crystal structures of MA was of an MA trimer [90,92,93] (Figure 7(a)). Also, 2D crystallography had revealed that MA can form a lattice composed of hexamers of trimers on PIP2-containing membranes [94]. More recent studies have demonstrated that mutating residues at the trimer interface results in a decrease of trimerization of MA, both *in vivo* and *in vitro* [95]. To test if MA trimerization influences PIP2 clustering, residues positioned at the MA trimer interface were mutated. A single mutant (T69D) and a double mutant (T69R/L74E) were tested (Figure 7(b)). As expected, no self-quenching of TF-PIP2 was observed for either of the trimer-defective mutants (Figure 7(c)). The same result was obtained under enhanced membrane binding conditions at 50 mM KCl (Figure S6). Similarly, the Q62R trimer-enhancement mutant [96] also did not promote PIP2 clustering (Figure 7(b) and (c)). Finally, an HIV-1 MA protein with 8 of its Lys and Arg residues switched with each other [13,30] (RK switch, Figure 7(c) and (d)) also caused no quenching. To rule out the possibility that the lack of induced PIP2 clustering was due to a loss of membrane binding, liposome pelleting assays were carried out for all four proteins. Only modest reductions in membrane binding were observed (Figure 7(c) bottom). The four mutations described above also were introduced into HIV-1 MACASP. All of the resulting proteins showed a 30% drop in TF-PIP2 fluorescence, like the wild-type protein. This observation indicates that the MA domain does not significantly enhance Gag multimerization. Taken together, all of these results lead to the conclusion that MA directs specific MA-PIP2 interactions required for promoting PIP2 cluster formation. The MA domain is necessary, but not sufficient. to induce PIP2 clustering.

### Multimerization-defective mutations in CA abrogate PIP2 clustering

**Figure 8.**
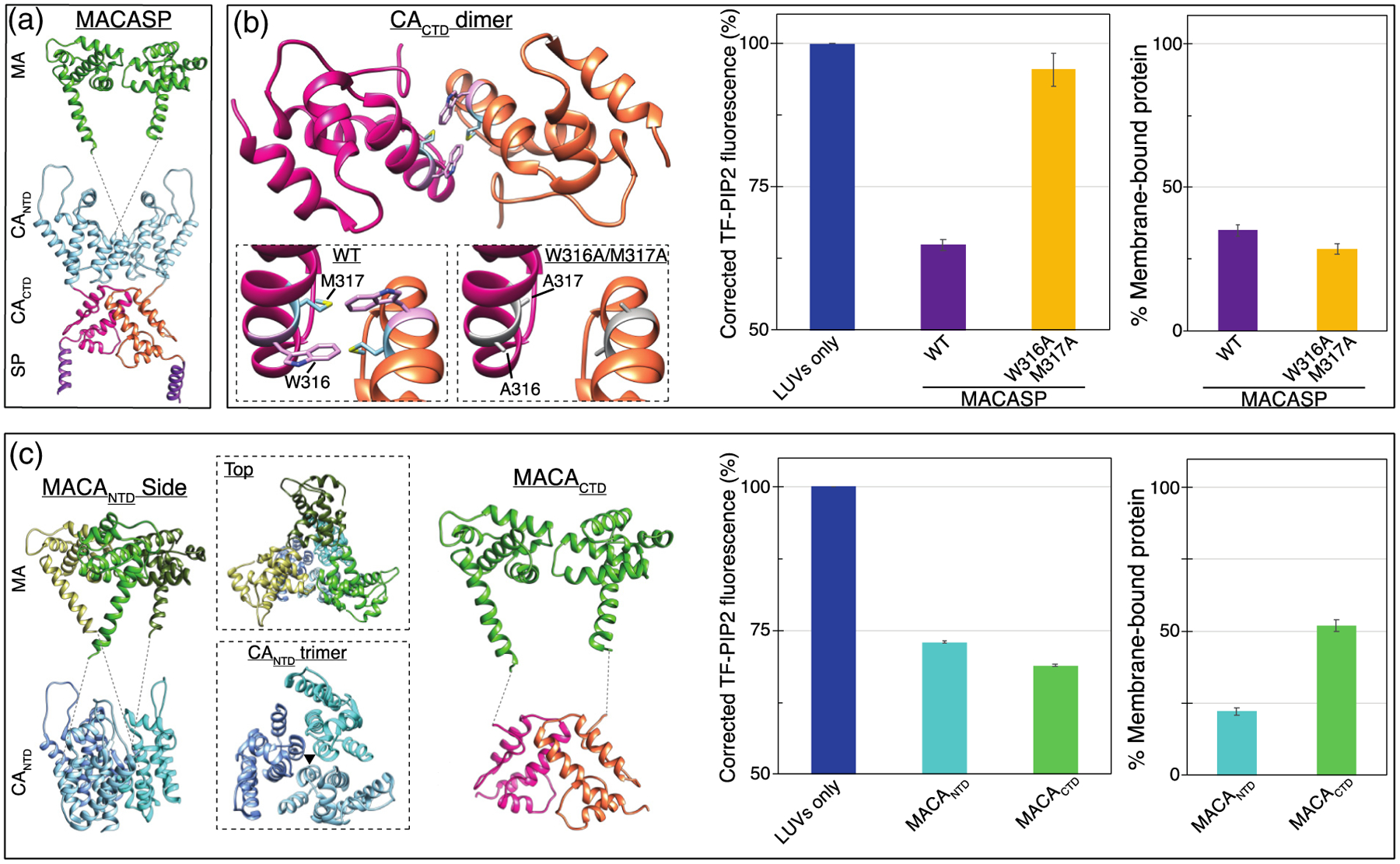
PIP2 clustering is dependent on ability of MA to multimerize via NTD and/or CTD interactions. (a) Model of MACASP dimer mediated by CA_CTD_-CA_CTD_ interaction. (b, left) CA_CTD_ dimer interface with amino acids W316 in pink and M317 in blue. Left inset is an enlargement of the dimer interface. Right inset shows W316A and M317A mutations which weaken the interface. (b, center) Effect of W316A/M317A mutations on PIP2 quenching by MACASP (b, right) and on membrane association of the protein. (c, left) Model of possible MACA_NTD_ trimer. Top inset shows a top down view of the MA trimer positioned above the CA_NTD_ trimer. Bottom inset shows the CA_NTD_ trimer, with the black triangle indicating the interface between monomers at helix two. The artificial MACA_CTD_ dimer. (c, center) Effect of MACA_NTD_ and MACA_CTD_ on PIP2 quenching (c, right) and membrane binding.

In all retroviruses multimerization of Gag results in the formation of an immature lattice and is based primarily on contacts between CA domains (Figure 8(a) and (b)). In HIV-1, homotypic dimerization of CA_CTD_ plays an essential role in multimerization [57–59] The CA dimerization-defective mutant W316A/M317A (WM Gag) leads to a 100-fold lower dimerization affinity [97,98] and severely impaired immature particle production *in vivo*. To further address the role of Gag multimerization in promoting PIP2 clustering, we examined MACASP with this WM mutation. The mutant protein barely induced PIP2 clustering, with only a 4% drop in TF-PIP2 fluorescence (Figure 8). This is consistent with a previous result comparing WT Gag to WM Gag [99]. We also tested HIV-1 MACA_NTD_ as well as MA fused with CA_CTD_ (termed MACA_CTD_) (Figure 8(c)). Both proteins were able to significantly induce PIP2 clusters to form, as evidenced by approximately a 30% drop in TF-PIP2 fluorescence (Figure 8(c)). In addition, a chimeric protein comprising RSV MA fused with HIV-1 CTD (termed RSV MACA_CTD_) induced a similar level of PIP2 clustering (Figure S7). For the HIV MACA_NTD_ construct, we predict that the multimerization occurring is a trimer, as evidenced by a recent cryoEM analysis of the HIV CA_NTD_ trimer interface, which appears critical for lattice formation [100]. These results suggest that either subdomain of CA is sufficient to multimerize HIV-1 Gag proteins that also have an MA domain.

To determine if a lattice was formed on the LUVs, we performed cryo-electron tomography on LUVs with bound MACASP protein. Reconstructed tomograms of virus particles clearly show a protein lattice [62,82,100–104], and so we hypothesized that if a lattice was forming on the membrane, it should be detectable using this method. Reconstructed tomograms clearly show a lipid bilayer with regions bound with protein (Figure S8). However, no clear lattice was observed. We interpret this to mean that in the LUV system, protein multimerization via dimer or trimer interfaces in CA are required for PIP2 clustering, but a lattice of tens or hundreds of proteins does not occur.

### SP-1 is not required for PIP2 cluster formation

**Figure 9.**
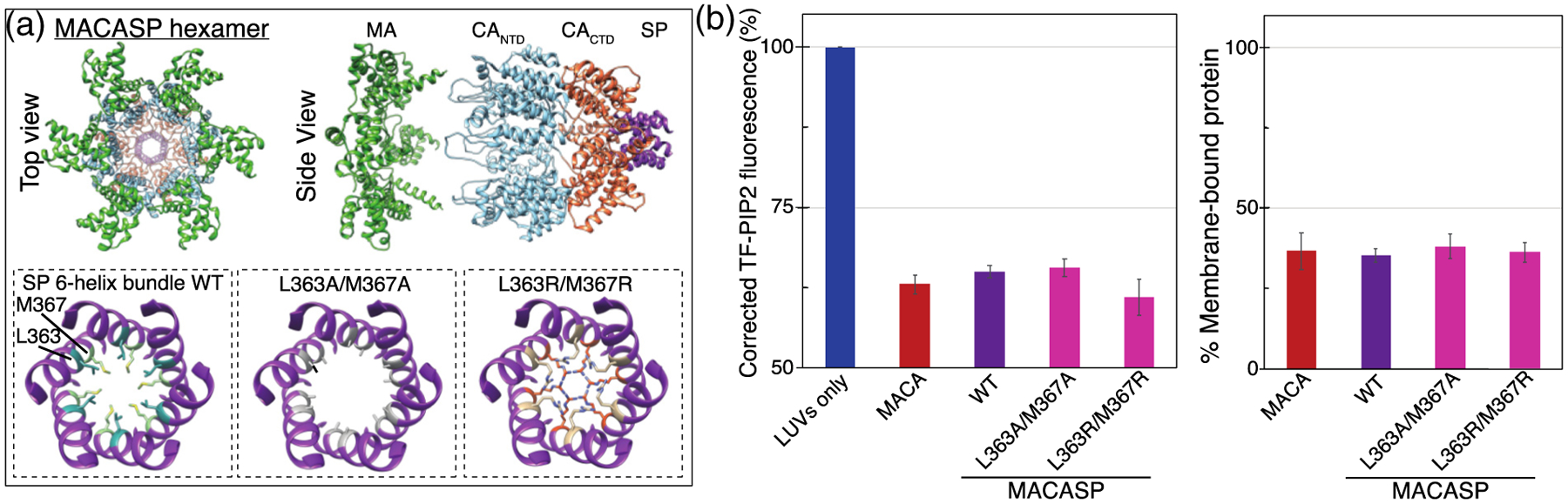
SP is not required for PIP2 cluster formation. (a) Top and side views of a model of the MACASP hexamer. Bottom left shows the structure of the 6HB (amino acids 355–371). Note that the 6HB includes the last nine residues of CA_CTD_ (355–363) and the first eight residues of SP (364–371). Side-chains of residues L363 (sea green) and M367 (light green) are shown. When mutated, these residues lead to a significant decrease in correct immature protein lattice assembly [59]. Bottom center shows the L363A/M367A mutation. Bottom right shows the L363R/M367R mutation. (b) Effect of MACA and MACASP without and with SP mutations on PIP2 quenching (left) and membrane binding (right).

During HIV-1 assembly, the SP1 peptide and a few immediately upstream residues in CA fold into a 6HB, which facilitates immature lattice formation. Mutations in SP1 [63], like similar mutations in the corresponding SP segment of RSV Gag [83], destroy the ability of Gag to assemble correctly, pointing to a critical role for this short domain in proper Gag multimerization. Nevertheless, we observed that HIV-1 SP1 is not required to induce lipid clustering. MACA induced PIP2 clustering to the same degree as MACASP (Figure 9). To further confirm this result, we mutated hydrophobic residues L363 in CA and M367 in SP1, which are located on the same side of the helix and mediate hydrophobic interactions between Gag molecules in the 6HB [105] (Figure 9(a)). These mutations, in the context of a Gag protein consisting of the MA-CA-SP1-NC domains of Gag, but with a deletion of the amino acid 16–99 MA membrane binding domain (typically referred to as ΔMA-CA-SP1-NC [106]), disrupts immature *in vitro* virus particle assembly [59]. Purified MACASP with either L363A/M367A or L363R/M367R showed membrane binding similar to HIV-1 MACASP WT at 100mM KCl (Figure 9(b)). Yet, both mutant proteins exhibited the same degree of induced PIP2 self-quenching (Figure 9(b)). Collectively, these results imply that SP1 is dispensable for the PIP2 clustering effect. Thus, the key driving force for clustering is the CA domain.

Based on the observation that SP1 is dispensable for PIP2 cluster formation, we examined the same WM mutation in the context of MACA. The mutant protein retained most of its ability to cause PIP2 clustering, and only a slight reduction in PIP2 self-quenching was observed (Figure S9). Thus, it seems that the WM mutation affected multimerization capacity only of HIV-1 MACASP, but not MACA. To explain these results, we speculate that the WM mutation mainly disrupts immature MACASP lattice formation, but not the primary interactions formed by the MACA protein.

### Multivalent cations and proteins can induce PIP2 clusters independently and additively

**Figure 10.**
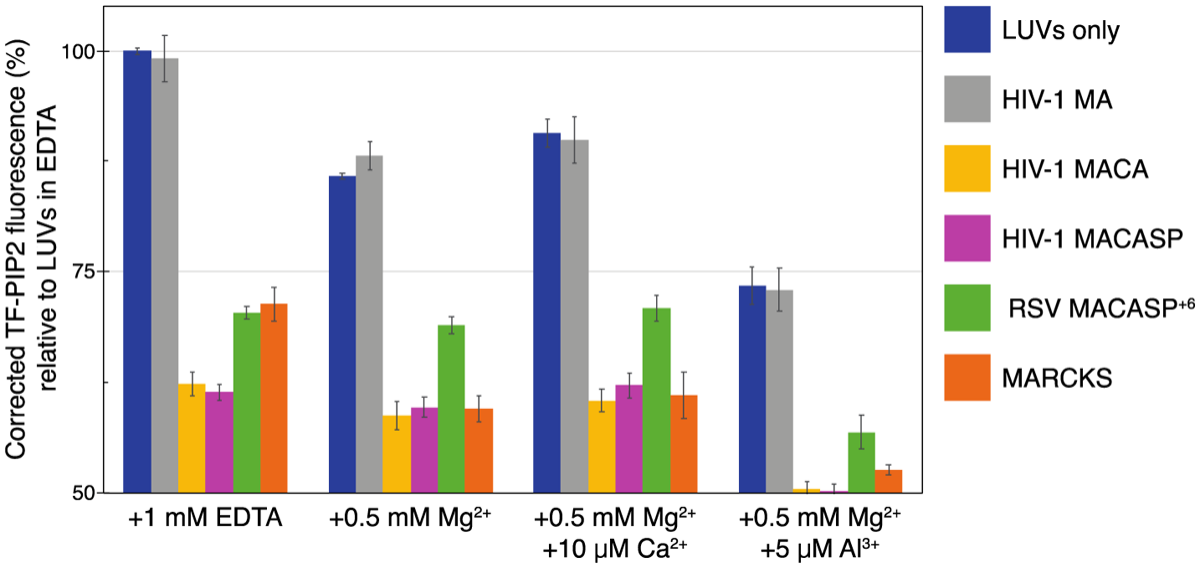
Proteins further promote PIP2 clustering of pre-existing multivalent cation-bridged PIP2. All LUVs were prepared in four different buffers that are based on 100 mM KCl, 20 mM HEPES, (pH 7.2), with additional EDTA or multivalent cations. PIP2 is free with 1 mM EDTA; PIP2 is modestly clustered with 0.5 mM Mg^2+^ or with 0.5 mM Mg^2+^ and 10 μM Ca^2+^, and PIP2 is strongly clustered with 0.5 mM Mg^2+^ and 5 μM Al^3+^. Note that lipids on both leaflets of the LUVs are exposed to the same buffer condition. Prior to protein addition, each protein/peptide was subjected to buffer exchange to match each buffer condition of LUVs. A total of 40 μl protein in each buffer was added to the outside of 160 μl LUVs in each buffer condition. TF-PIP2 fluorescence of LUVs mixed with buffer containing EDTA was maximum, set to 100%, while TF-PIP2 fluorescence of LUVs prepared with multivalent cations and/or mixed with protein or peptide was converted to the corresponding percentage. Note that for protein-induced PIP2 clustering, *Y*-axis is only shown from 50% to 100%. Each bar in the graph represents the average TF-PIP2 fluorescence % over the 1, 2, and 3 min time points post-mixing. Each quenching assay was performed at least three times; error bars show standard deviations from the means.

The results presented above demonstrate that both multivalent cations and certain membrane-binding proteins independently can induce PIP2 to form clusters in model membranes. The protein experiments were purposely carried out in 1 mM EDTA, a non-physiological condition, in order to separate protein and metal ion effects on PIP2. To examine protein-induced clustering in a more biologically relevant setting, we tested whether proteins could promote further PIP2 clustering in the presence of multivalent cations (Figure 10). HIV-1 MACA, HIV-1 MACASP, RSV MACASP^+6^, and MARCKS(ED) were chosen for this analysis. The same quenching assay was performed in four parallel buffers: LUVs and protein mixtures in EDTA to eliminate multivalent cation effects (free PIP2); 0.5 mM Mg^2+^ to mimic a cytosol resting state (moderately clustered PIP2); 0.5 mM Mg^2+^ and 10 μM Ca^2+^ to mimic a transient calcium influx (moderately clustered PIP2); and 0.5 mM Mg^2+^ and 5 μM Al^3+^ to maximize clustering (strongly clustered PIP2) (Figure 10). All of the retroviral Gag mimics tested, as well as the well-studied MARCKS (ED), further promoted PIP2 clustering, as the TF-PIP2 fluorescence dropped lower than that in the respective LUV with EDTA samples. In summary, these results demonstrate that multivalent cation-bridged PIP2 clusters do not abolish proteins’ capacity to further promote PIP2 clustering. Thus, the effects of multivalent cations and protein appear to be independent and additive.

## Discussion

We reported previously that PIP2 can form multivalent cation-bridged PIP2 clusters in model membranes that mimic the composition of the inner leaflet of the PM. We now show that myristoylated HIV-1 MA preferentially binds to such pre-existing PIP2 clusters, compared with free PIP2, and that this preference requires the N-terminal myristoyl modification. Binding of Gag proteins promotes further PIP2 clustering, and this process relies on specific interactions between the MA domain and PIP2. Previous work had suggested a model that instead of targeting pre-existing lipid domains, Gag generates nanodomains enriched in PIP2/Chol at viral assembly sites [11,99]. Here, we present an updated model that HIV-1 Gag not only preferentially targets pre-existing PIP2 clusters induced by multivalent cations, but also further sequesters PIP2 as Gag assembles. Taken together, our study provides direct evidence for a model that could mechanistically explain why HIV-1 viral membranes are enriched in PIP2. In this model, HIV-1 Gag targets pre-existing PIP2-rich domains as assembly sites, and then stabilizes and further enriches PIP2 as Gag multimerization leads to formation of the immature Gag lattice at the site of virus budding.

The inferences above are based on two assays, each being robust but having its own limitations. We have extensive experience in preparation of GUVs with known lipid compositions [107]. Quantitation of fluorescent protein binding is straightforward with confocal microscopy. However, the composition of a single individual GUV cannot be determined and does vary to some extent, which likely accounts in part for the spread in standard deviations in protein fluorescence values. Therefore, it is imperative to make many independent measurements (in our experiments over 30 for each combination of protein and GUV) and then acquire statistics. Also, while it is straightforward to quantitatively compare binding for different proteins, it is not possible to derive binding constants from such measurements, especially for proteins that can multimerize on the membrane. In our hands, under all conditions tested, fluorescent protein binding to GUVs appeared uniform, as did multivalent cation-induced PIP2 clustering, implying that the inferred lipid clusters are below optical resolution.

Self-quenching of fluorescent lipid probes, like the technique of FRET, reports on very local molecular proximity. In our experiments, PIP2 was labeled on the sn-2 acyl chain with a BODIPY analogue carrying the trade name TopFluor. We established previously that this fluorescent analogue, as well as a different chain-labeled PIP2, TMR-PIP2, behave like natural PIP2 [67]. In the experiments reported here with large (~100 nm) unilamellar vesicles, the fluorescent lipid was always present with an excess of the natural, brain-derived PIP2, in order to more closely mimic a biologically relevant PM. As shown previously [67], all of the other PIPs co-cluster with PIP2, but PI and other glycerophospholipids do not, thus leading to the inference that clustering is mediated by one or more phosphate groups on the inositol ring. The length and saturation of the *sn1* fatty acyl chain do not play a measurable role in clustering [67]. Being measurements in cuvettes, the fluorescence values reported here, with and without different proteins, are precise, as can be seen for example by the small standard deviations expressed as error bars in the data. Therefore, even a small degree of quenching caused by an added protein is indicative of a degree of clustering. However, a limitation in interpreting our results is that it is not possible to infer from our quenching values the actual distances between lipid molecules. One can say with confidence only that a higher level of quenching implies a greater density of labeled PIP2. The actual density, the possible presence of other lipids in the cluster, and the size and molecular structure of the cluster remain unknown.

To assess both metal cation-induced and protein-induced PIP2 clustering in LUVs, TF-PIP2 fluorescence was converted to a percentage relative to the maximum fluorescence in the presence of 1 mM EDTA. When present, metal ions were on both sides of the membrane whereas the protein was present only on the outside, under the standard assumption that the vesicles were not leaky. Thus, the strongest metal ion quenching by 100 μM Al^3+^, resulting in a 70% loss in fluorescence, corresponds approximately to the strongest protein-induced quenching by HIV-1 MACASP or MARCKS ED, resulting in a 35% loss in fluorescence. While Al^3+^ is presumed to be non-physiological, 0.5 mM Mg^2+^, which is approximately the free concentration in cytosols of mammalian cells [108], also leads to substantial self-quenching, with an observed 12% loss in total fluorescence or 6% for each leaflet. And in the presence of an additional 10 μM Ca^2+^, corresponding to a local transient spike of calcium influx, the self-quenching is slightly augmented. In summary, the degree of PIP2 clustering induced by proteins can be of similar magnitude as that by multivalent cations, depending on the type of protein and its local concentration.

The fact that PIP2 clustering can be manipulated by the presence or absence of multivalent metal ions allowed us to address the relative preference of membrane binding proteins for clustered PIP2 *versus* freely dispersed PIP2. A striking and unanticipated finding was that the myristoyl group on proteins appears to play a role in this process. Thus, myristoylated HIV-1 MA showed a strong preference for metal ion-induced clusters of PIP2, while unmyristoylated MA preferred to bind to free PIP2. Similarly, in the artificially constructed mNG protein with a basic N-terminal tail, the myristoylated version preferred clustered PIP2 but the unmyristoylated version did not. These findings could be explained, at least in part, by the stronger protein binding mediated by the fatty acyl modification. We hypothesize that only strong membrane binding proteins can compete off PIP2-bound multivalent cations to gain the access to PIP2 headgroups, while weaker membrane binding proteins have access only to free PIP2 headgroups. This model is supported by the observation that RSV MA-CcmK4, the non-myristoylated hexamerized version of MA that is known to bind very strongly to membranes, also prefers PIP2 in clusters. By contrast, monomeric RSV MA prefers to bind to free PIP2. Other than the RSV [25,29] and HIV-1 MA domains, there is no conclusive data on specific PIP2 binding by other retroviral MA domains, although all MA domains would bind strongly with PIP2 via electrostatic interactions. However, we cannot eliminate the additional possibility that PIP2 clusters have a unique acyl chain environment that is more favorable to myristate insertion than the bulk membrane.

The phospholipase Cδ1 PH domain probably is the best known PIP2 binding module, and when fused to fluorescent proteins, is used commonly as a marker for the PM. We found that the membrane binding of PH-mNG is strongly inhibited in cation-bridged PIP2 clusters, being decreased two-fold in Ca^2+^ buffer and almost 10-fold in Al^3+^ buffer. In addition, the inhibitory effect of metal ions is consistent with the report that Ca^2+^ affects the tilt angle of the PIP2 headgroup, preventing optimal interaction with the PH binding pocket [74]. This PH behavior contrasts with that of another PM binding module, the unstructured basic domain of the MARCKS protein (MARCKS(ED)), which binds equally to clustered and dispersed PIP2. Thus, PH may serve as a suitable probe only for free PIP2. The different behavior of these two cellular protein domains highlights the importance of the PIP2 lateral distribution in determining protein interaction. In cells, PIP2 is likely to exist in both free and clustered forms. Hence, a local change in multivalent cation concentration, such as a transient calcium influx, could quickly modulate protein binding.

Despite several clues, the mechanisms underlying the ability of proteins to cause PIP2 clustering remain incompletely understood. While interaction of the protein with PIP2 in membranes is necessary, it is clearly not sufficient. The strength of membrane binding also appears not to be directly related to induction of PIP2 clustering. Thus, for all proteins tested, PIP2 self-quenching was similar at 100 mM and 50 mM KCl, even though protein binding at the lower salt was increased. For PIP2 clustering to occur, the interacting protein apparently must have some ability to form multimeric complexes, as best evidenced by the several HIV-1 Gag proteins that we tested. And yet, while purified MA can form trimers in solution and in crystals [90,93], and myristoylated HIV-1 MA can form hexamers of trimers on PIP2-containing membranes [88,94], neither non-myristoylated nor myristoylated MA induced PIP2 clustering in our assays. Even MA carrying the trimer-enhancement mutation Q62R failed to induce clustering. These results could be explained by two models. In the first, the MA–MA protein interactions are too weak to support stable clustering of PIP2. In the second, three lipid molecules brought together by an MA trimer are insufficient to form a cluster that shows self-quenching of acyl chain labeled PIP2.

The strong and reproducible phospholipase Cδ1 PH domain (PH)-induced PIP2 clustering is difficult to understood. However, our result here is consistent with a similar observation from a previous study [69]. One possibility is that the PH domain can oligomerize on membranes. Alternatively, perhaps each PH actually binds to multiple PIP2, contrary to the 1:1 stoichiometry inferred from structural studies [109]. The facts that, on the one hand PH preferentially binds to free PIP2, but on the other PH can promote PIP2 clustering, remain to be explained in a mechanistic way.

We made the surprising observation that the 6His tag that is commonly used for purifying HIV-1 MA [86–88] can induce significant PIP2 clustering. This artifact was confirmed by testing purified myristoylated HIV-1 MA, non-myristoylated HIV-1 MA, and RSV MA– all with or without a C-terminal 6His tag. Not only did the tagged forms of all three proteins cause PIP2 clustering, but they also exhibited stronger membrane binding. We speculate that the consecutive His residues are sufficient to pull multiple PIP2 molecules together via electrostatic interactions. While there are reported artifacts introduced by 6His tag in other proteins, to our knowledge, this is the first evidence that the tag can significantly influence HIV-1 MA membrane binding properties. It will be critical in future studies by researchers in this field to be aware of this important finding.

In contrast to MA itself, Gag proteins that include the MA domain as well as downstream domains capable of multimerizing, caused strong PIP2 clustering. Thus, for example, MACASP as well as MACA led to dramatic self-quenching of the acyl chain-labeled PIP2 reporter. Since only the former protein includes the SP sequence that forms a 6HB in the Gag lattice, we expected MACASP to be more potent than MACA in the quenching assay. However, the two proteins behaved the same. Similarly, we expected the MA trimerization-defective mutations in the context of MACASP to show less quenching, but surprisingly they did not behave differently than the wild-type MACASP, suggesting that the MA trimer interface interactions are weak in the context of other Gag–Gag interactions. Among all the MA mutations that we tested, only the K30E/K32E mutant in the context of HIV-1 MACASP failed to induce any PIP2 clustering, even though it still showed membrane binding that was sufficient for other proteins to induce clustering. These several results indicate that the predominant role of the MA domain in inducing PIP2 clustering is to provide specific interaction with PIP2, while CA is the key player in promoting PIP2 clustering by Gag proteins.

To try to dissect what features of HIV-1 CA are the most important for the PIP2 clustering effect, we generated a series of mutant forms of the MACA and MACASP proteins. The mutations were designed to eliminate or reduce various protein–protein interactions known to occur in the Gag lattice. The best known such mutation is the W316A/M317A (here called WM), which disrupts the CA_CTD_-CA_CTD_ dimer interface at hexamer-hexamer contacts in the immature lattice [57–59]. This mutation led to a complete loss in PIP2 clustering in the context of MACASP. However, the same mutation caused only a slight decrease in PIP2 clustering in the context of MACA. To explain this result, we hypothesize that these two Gag proteins form different lateral protein contacts on membranes *in vitro*. The longer MACASP is likely to form predominantly immature dimer interfaces and perhaps immature hexamers, since the SP region could create a 6HB. By contrast, the shorter MACA can form both an immature or a mature-like lattice, as evidenced by cryo-electron tomography analysis of the lattice of virus-like particles from cells expressing Gag cleavage site mutations [102].

Both HIV-1 proteins that include MA and only a part of CA, i.e. MACA_NTD_ and MA-CA_CTD_, can promote clustering. The latter is an artificial construct that joins two sequences that are not neighbors in Gag. This implies that both CA_NTD_ and CA_CTD_ can themselves form multimeric complexes that are sufficient to sequester multiple PIP2 molecules. According to published structures, within the immature hexameric Gag lattice, HIV-1 CA_NTD_ forms the inner ring of the hexamers as well as a trimeric and dimeric interface between hexamers. The HIV-1 CA_CTD_ also forms a hexamer as well as a dimeric interface between hexamers. It is not clear from our data for MACA_NTD_ if one of the three interfaces is more important than the others for enhancing PIP2 clustering. We did not test the myristoylated forms of HIV-1 MACASP or of Gag itself, since those proteins could not be purified at sufficient concentrations in soluble form. Overall, the ability of both MACA_NTD_ and MA-CA_CTD_ to promote PIP2 clustering confirms the critical role of CA in enriching local PIP2 at assembly sites. This multimerization capability might also explain why HIV-1 Gag preferentially binds to clustered PIP2, since several Gag molecules in a complex would exhibit strong membrane binding affinity, and thus be able to displace or bind with multivalent metal ions from preformed clusters.

In summary, this study demonstrates that both multivalent cations and proteins can induce PIP2 to form clusters, independently and additively, in a biologically relevant lipid environment. Protein is able to recognize and utilize the lateral organization of PIP2 induced by multivalent cations, and further reorganize PIP2 lateral distribution upon protein multimerization. In cells, multivalent cation-induced PIP2 clusters might serve as a regulatory platform for protein–membrane binding. Depending on their intrinsic properties, proteins like HIV-1 Gag could have access predominantly to locally concentrated PIP2 in clusters, or to free PIP2 headgroups when this lipid is dispersed. Once bound to membranes, proteins that either contain a long stretch of basic charges like MARCKS, or that have multimerization ability like Gag, might efficiently sequester more PIP2. Our study provides a mechanistic model to explain PIP2 enrichment in the HIV-1 membrane. In this model, Gag assembles at pre-existing PIP2-rich domains because of the preferential binding of the myristoylated MA domain to clustered PIP2. Then, as Gag multimerizes to form the immature lattice, it causes further PIP2 clustering before virus budding from the cell.

## Materials and Methods

### DNA cloning and protein purification

DNA constructs used for non-labeled protein purification were cloned straight into pSUMO or pET3xc vector using Integrated Device Technology gene block techniques. For fluorescently labeled proteins, mNG was amplified from pHisII 6H-mNG, and ligated into pSUMO or pET3xc vectors. All proteins were purified using standard bacterial expression and affinity column techniques. In brief, *E. coli* BL21 cultures were grown at 37 °C to an optical density at 600 nm of 0.6. For myristoylated proteins, DNA constructs in the pET plasmid. and another plasmid that expresses yeast N-terminal myristoyl transferase, were co-transformed into BL21. For myristoylated proteins, myristic acid (10 mg/L, Sigma) was added to BL21 cultures 1 h before induction [86]. IPTG was added to a final concentration of 0.5 mM for induction. Induced cells were harvested 4–6 h post-induction.

The cells were resuspended in lysis buffer (20 mM Tris (pH 8), 500 mM NaCl, 2 mM tris(2-carboxyethyl)-phosphine (TCEP), and 2 mM phenylmethylsulfonyl fluoride (PMSF)) and lysed by sonication. After ultracentrifugation in a TLA-110 Beckman rotor at 90,000 rpm for 45 min, the supernatant was collected. Then, supernatants were treated with polyethyleneimine and centrifuged at 10,000 rpm in a Sorvall 600 rotor at 4 °C to remove nucleic acid. To the supernatant, ammonium sulfate was added until precipitated protein was observed by eye, usually in the range of 20%–30%, followed by centrifugation. The pellet was resuspended in binding buffer (20 mM Tris–HCl (pH 8), 100 mM NaCl, and 2 mM TCEP) and further purified by desalting chromatography (HiPrep 26/10 desalting; GE Healthcare) and Ni^2+^ affinity chromatography. Following the first round of Ni^2+^ chromatography, eluted proteins were dialyzed against buffer (20 mM Tris–HCl (pH 8), 500 mM NaCl, and 1 mM TCEP). Following the first round of Ni^2+^ chromatography, eluted proteins were dialyzed against buffer (20 mM Tris–HCl (pH 8), 500 mM NaCl, and 1 mM TCEP). Proteins without the 6His tag were purified by desalting chromatography (HiPrep 26/10 desalting; GE Healthcare) and ion exchange chromatography (HiTrap SP FF; GE Healthcare) directly after polyethyleneimine and ammonium sulfate treatment. For proteins expressed in pSUMO vectors, the dialysis was performed in the presence of approximately 300 μg of ULP1 protease to cleave off the SUMO tag. The ULP1 protease and SUMO tag were removed by a second around of Ni^2+^ affinity chromatography [110]. Purified protein at 2 to 10 mg/ml was flash frozen in aliquots and stored at −80 °C. The final protein preparation had an A260/A280 ratio of 0.58 to 0.59, indicating the absence of nucleic acid. All proteins had a purity of approximately 90% after affinity column purification, as judged from stained SDS polyacrylamide gels.

### Phospholipids and fluorescent probes

The following lipids were purchased from Avanti Polar Lipids (Alabaster, AL): 1-palmitoyl-2-oleoyl-sn-glycero-3-phosphoethanolamine (POPE), 1-palmitoyl-2-oleoyl-sn-glycero-3-phospho-L-serine (POPS), L-a-phosphatidylinositol-4,5-bisphosphate (bovine brain-PI(4,5) P2), fluorescently labeled TopFluor (TF)- PI(4,5)P2 (1-oleoyl-2-{6-[4-(dipyrrometheneboron difluoride)butanoyl] amino} hexanoyl-sn-glycero-3-phosphoinositol-4,5-bisphosphate), TMR-PI (4,5)P2 (1-oleoyl-2-(6-((4,4-difluoro-1,3-dimethyl-5-(4-methoxyphenyl)-4- bora-3a,4a-diaza-s-indacene-2-propionyl)amino)hexanoyl)-sn-glycero-3- phosphoinositol-4.5-bisphosphate. Cholesterol was purchased from Nu-Chek Prep (Elysian, MN). Cholesterol stock solutions were prepared by standard gravimetric procedures to within 0.2% error. Concentrations of all phospholipid stocks were determined to 1% error by inorganic phosphate assay [111]. The working stocks of TF- and TMR-labeled PIP2 were prepared in chloroform:methanol:H_2_O = 20:9:1. Fluorescent probe extinction coefficients were obtained from lot certificates of analysis: 97,000 M^−1^ cm^−1^ at 496 nm for TF and 56,000 M^−1^ cm^−1^ at 544 nm for TMR. Probe concentrations were determined in methanol by absorption spectroscopy using an HP 8452A spectrophotometer (Hewlett-Packard, Palo Alto, CA). Lipid purity of 99.5% was confirmed by thin-layer chromatography (TLC). TLC was performed on washed, activated silica gel plates (Alltech, Deerfield, IL) developed with chloroform:methanol:water = 65:25:4 for most phospholipids and for cholesterol with petroleum ether:diethyl ether:chloroform = 7:3:3. TLC plates for PIP2 were pre-run with 10% K_2_C_2_O_4_ + 2 mM EDTA and then activated at 100 °C for 30 min before use. TLC plates for PIP2 were developed with chloroform: methanol:4 N NH_4_OH = 45:35:10 [67].

### Buffer preparation and metal ion measurement

All buffers used were based on 100 mM KCl, 20 mM HEPES (pH 7.2). “Pure buffers” were prepared with KCl (99.999%; Sigma-Aldrich or ACROS (Geel, Belgium)), HEPES (99.5%; Sigma-Aldrich), and stored in Teflon fluorinated ethylene propylene bottles (Nalgene). Water was purified to 18.2 MΩ by passage through a Barnstead MicroPure system (Thermo Fisher, Waltham, MA). Micromolar levels of Al^3+^, Ca^2+^, and Mg^2+^ were prepared from 100 mM stock solutions stored at pH 2–3. These stocks were made with aluminum chloride (99.999%), calcium chloride (99%), and magnesium chloride hexahydrate (99%), all from Sigma-Aldrich. Disodium EDTA (99%; Sigma-Aldrich) was prepared and stored as a 500 mM stock solution at pH 7.2. Ion concentrations and purities of all stocks and freshly prepared buffers were confirmed by inductively coupled plasma optical emission spectroscopy (ICP-OES) [112] at the Cornell Nutrient Analysis Laboratory using Spectro Arcos ICP-OES.

### LUV preparation

A total of 250 nmol lipid mixtures was dispensed into each borosilicate culture tube using glass syringes (Hamilton, Reno, NC). LUVs were prepared using rapid solvent exchange in order to reduce lipid demixing artifacts, followed by extrusion through polycarbonate filters 21 times [113]. Samples were sealed under argon at a final lipid concentration of 0.5 mM.

### Self-quenching

Purified proteins were subject to buffer exchange and concentrating using centrifugal filter columns (Amicon Ultra 0.5ml-10KD; Millipore Sigma). The final protein was in buffers matching the corresponding LUV buffers, in 100 mM KCl, 20 mM HEPES (pH 7.2), with or without additional EDTA or multivalent cations. A volume of 160 μl of 0.5 mM LUVs and 40 μl of purified protein and buffers were mixed in the microcuvette (115F- Micro cells, 10 × 2-mm light path; Hellma Analytics) to reach a total volume of 200 μl at room temperature. The final protein–membrane mixture was 400 μM lipids and 20 μM proteins. If PIP2 was 2 mol% of total lipids, a calculated 4 μM PIP2 was exposed to proteins on the outer leaflet. Fluorescence was collected on a Hitachi F-7000 FL spectrofluorimeter (Hitachi High Technologies America, Schaumburg, IL) at 23 °C. Wavelengths used for self-quenching studies were (ex/em) as follows: TF (485/515 nm) and light scattering (440/420 nm). Data were collected with slits for ex/em = 2.5/2.5 nm and a 10-s integration time. TF-PIP2 fluorescence under each condition was converted to percentage relative to the maximum fluorescence of TF-PIP2 in the LUVs only sample in the buffer containing 1 mM EDTA.

### Liposome pelleting assay

After self-quenching and FRET measurements, protein–LUV mixtures were ultracentrifuged at 75,000 rpm in a TLA-100 (Beckman) rotor for 15 min at 4 °C, which is sufficient to bring down all the LUVs to the pellet (data not shown). The supernatant was removed and the pellet was resuspended and subjected to SDS-PAGE analysis [83]. Gels were Coomassie blue stained overnight and destained, and band intensity was determined by densitometry analysis using ImageQuant software 5.2 [107].

### GUV preparation and confocal imaging

A total of 250 nmol total lipid containing 0.01 mol% 18:1 DiI was mixed in chloroform, partially dried to a thin film in a culture tube using a rotary evaporator, and then dried with heating at 55 °C under high vacuum for 1.5 h. The thin dry film was then hydrated with wet N_2_ gas at 55 °C for 30 min. Lipid films were further hydrated with prewarmed ~225 mM sucrose buffer (Fisher Scientific) and incubated at 55 °C for 2 h. GUVs formed as the sample were cooled over 10 h to room temperature (23 °C) [107]. GUVs were harvested into pure buffer 100 mM KCl, 20 mM HEPES (pH 7.2), with or without additional EDTA or multivalent cations. Harvested GUVs were then incubated in each buffer condition for at least 5 h before imaging. All buffers were osmotically balanced, confirmed by measurements using an osmometer (model No. 5004; Precision Systems, Natick, MA). An Eclipse C2+ Confocal Microscope (Nikon Instruments) with a 60×/1.2 NA water immersion objective was used for GUV imaging at 23 °C. Sample chambers for observation consisted of a No. 1.5 coverslip and traditional microscope slide separated with a silicone spacer (Sigma-Aldrich) of 0.25-mm thickness. Both microscope slide and coverslip used for GUV–protein binding assays were pre-coated with 0.03% Casein blocking buffer in PBS overnight, and then were rinsed with water and dried before imaging. This blocking procedure prevents mNG-labeled protein adsorption onto the glass. Fluorescence signals were quantified in Fiji using the plot profile function. Line scan analysis was performed across the perimeter of each GUV to obtain an average fluorescence intensity on the membrane, with background fluorescence subtracted. Two separate line scans per GUV were analyzed. No fewer than 30 GUVs that contained 2mol % PIP2, and no fewer than 15 GUVs that did not contain PIP2 were analyzed for each protein under each buffer condition (see Figure 2).

### Cryo-electron tomography of protein bound LUVs

LUVs (POPE/POPS/Chol/PIP2 at mole ratios of 32/30/36/2) and MACASP protein were mixed at the same ratio as in the pelleting reaction but at five times higher concentrations (2000 μM lipid, 100 μM MACASP protein). Final buffer concentrations for the binding reactions were 100 mM KCl, 20 mM HEPES (pH 7.2), 1 mM EDTA. Binding reactions were mixed 1:1 with Protein A conjugated gold 10 nm (Aurion), and 3 μl were spotted onto glow discharged (45 s, 20 mA) 2/2–3C C-flat grids. Samples were vitrified in liquid ethane using a Vitrobot Mark 4 plunger (blot time of 3 s and a blot force of 0). Samples were stored under liquid nitrogen until imaging.

Imaging was performed at 200 kV on a Thermo Fisher Talos Arctica TEM equipped with a Gatan K3 direct detector and BioContinuum imaging filter using the SerialEM software package [114]. Tilt series were acquired using a dose symmetric tilt scheme [115] from −60° to 60° at 3° steps. The magnification was 63,000×, with a pixel size of 1.25 Å/pixel. The total dose was ~ 140 e ^−^/Å/s. Tomograms were reconstructed using IMOD [116].

## Supplementary Material

Supplementary data

## Abbreviations used:

HIV-1: human immunodeficiency virus
MA: matrix protein
myr: myristoylation
GUV: giant unilamellar vesicle
LUV: large unilamellar vesicle
PM: plasma membrane
HBR: highly basic region
PS: phosphatidylserine
CT: cytoplasmic tail
Env: envelope
6HB: six-helix bundle
FRET: Förster resonance energy transfer
PH: pleckstrin homology
mNG: monomeric Neon Green
RSV: Rous sarcoma virus
TLC: thin-layer chromatography

## References

[1] Dick RA, Zadrozny KK, Xu C, Schur FKM, Lyddon TD, Ricana CL, Wagner JM, Perilla JR, , (2018). Inositol phosphates are assembly co-factors for HIV-1. Nature, 10.1038/s41586-018-0396-4. PMC6242333 30069050

[2] Dick RA, Mallery DL, Vogt VM, James LC, (2018). IP6 regulation of HIV capsid assembly, stability, and uncoating. Viruses, 10, 640, 10.3390/v10110640. PMC6267275 30445742

[3] Mallery DL, Rifat Faysal KM, Kleinpeter A, Freed EO, Saiardi A, James LC, Wilson MSC, Vaysburd M, , (2019). Cellular IP 6 levels limit HIV production while viruses that cannot efficiently package IP 6 are attenuated for infection and replication correspondence article cellular IP 6 levels limit HIV production while viruses that cannot efficiently package IP 6 are attenuated for infection and replication. Cell Rep., 29, 3983–3996, 10.1016/j.celrep.2019.11.050. 31851928 PMC6931105

[4] Sundquist WI, Kräusslich HG, (2012). HIV-1 assembly, budding, and maturation. Cold Spring Harb. Perspect. Med, 2, 1–25, 10.1101/cshperspect.a006924. PMC3385941 22762019

[5] Briggs JAG, Riches JD, Glass B, Bartonova V, Zanetti G, Kräusslich HG, (2009). Structure and assembly of immature HIV. Proc. Natl. Acad. Sci, 10.1073/pnas.0903535106. PMC2700151 19549863

[6] Chan R, Uchil PD, Jin J, Shui G, Ott DE, Mothes W, Wenk MR, (2008). Retroviruses human immunodeficiency virus and murine leukemia virus are enriched in phosphoinositides. J. Virol, 82, 11228–11238, 10.1128/JVI.00981-08. 18799574 PMC2573248

[7] Mücksch F, Citir M, Lüchtenborg C, Glass B, Traynor-Kaplan A, Schultz C, Brügger B, Kräusslich HG, (2019). Quantification of phosphoinositides reveals strong enrichment of PIP2 in HIV-1 compared to producer cell membranes. Sci. Rep, 10.1038/s41598-019-53939-z. PMC6881329 31776383

[8] Ono A, Ablan SD, Lockett SJ, Nagashima K, Freed EO, (2004). Phosphatidylinositol (4,5) bisphosphate regulates HIV-1 Gag targeting to the plasma membrane. Proc. Natl. Acad. Sci, 101, 14889–14894, 10.1073/pnas.0405596101. 15465916 PMC522033

[9] Chukkapalli V, Hogue IB, Boyko V, Hu WS, Ono A, , (2007). Interaction between the human immunodeficiency virus type 1 Gag matrix domain and phosphatidylinositol-(4,5)-bisphosphate is essential for efficient Gag membrane binding. J. Virol, 82, 2405–2417, 10.1128/jvi.01614-07. 18094158 PMC2258911

[10] Mücksch F, Laketa V, Müller B, Schultz C, Kräusslich H-G, (2017). Synchronized HIV assembly by tunable PIP2 changes reveals PIP2 requirement for stable Gag anchoring. Elife, 6, 10.7554/eLife.25287. PMC5495570 28574338

[11] Favard C, Chojnacki J, Merida P, Yandrapalli N, Mak J, Eggeling C, Muriaux D, (2019). HIV-1 Gag specifically restricts PI(4,5)P2 and cholesterol mobility in living cells creating a nanodomain platform for virus assembly. Sci. Adv, 10.1126/sciadv.aaw8651. PMC6774721 31616784

[12] Dick RA, Goh SL, Feigenson GW, Vogt VM, (2012). HIV-1 Gag protein can sense the cholesterol and acyl chain environment in model membranes. Proc. Natl. Acad. Sci. U. S. A, 10.1073/pnas.1209408109. PMC3503231 23010924

[13] Olety B, Ono A, (2014). Roles played by acidic lipids in HIV-1 Gag membrane binding. Virus Res., 10.1016/j.virusres.2014.06.015. PMC4252750 24998886

[14] Mercredi PY, Bucca N, Loeliger B, Gaines CR, Mehta M, Bhargava P, Tedbury PR, Charlier L, , (2016). Structural and molecular determinants of membrane binding by the HIV-1 matrix protein. J. Mol. Biol, 10.1016/j.jmb.2016.03.005. PMC4836608 26992353

[15] Waheed AA, Freed EO, (2010). The role of lipids in retrovirus replication. Viruses., 2, 1146–1180, 10.3390/v2051146. 20740061 PMC2927015

[16] Resh M, (1999). Fatty acylation of proteins: new insights into membrane targeting of myristylated and palmitoylated proteins. Biochim. Biophys. Acta - Mol. Cell Res, 1451, 1–16, 10.1016/S0167-4889(99)00075-0. 10446384

[17] Bouamr F, Scarlata S, Carter C, (2003). Role of myristylation in HIV-1 Gag assembly. Biochemistry, 10.1021/bi020692z. 12767222

[18] Barros M, Heinrich F, Datta SAK, Rein A, Karageorgos I, Nanda H, Lösche M, (2016). Membrane binding of HIV-1 matrix protein: dependence on bilayer composition and protein lipidation. J. Virol, 90, 4544–4555, 10.1128/JVI.02820-15. 26912608 PMC4836311

[19] Li H, Dou J, Ding L, Spearman P, (2007). Myristoylation is required for human immunodeficiency virus type 1 Gag–Gag multimerization in mammalian cells. J. Virol, 81, 12899–12910, 10.1128/jvi.01280-07. 17881447 PMC2169113

[20] Provitera P, El-Maghrabi R, Scarlata S, (2006). The effect of HIV-1 Gag myristoylation on membrane binding. Biophys. Chem, 119, 23–32, 10.1016/j.bpc.2005.08.008. 16183191

[21] Chukkapalli V, Ono A, (2011). Molecular determinants that regulate plasma membrane association of HIV-1 Gag. J. Mol. Biol, 410, 512–524, 10.1016/j.jmb.2011.04.015. 21762797 PMC3139151

[22] Cannon PM, Matthews S, Clark N, Byles ED, Iourin O, Hockley DJ, Kingsman SM, Kingsman AJ, (1997). Structure-function studies of the human immunodeficiency virus type 1 matrix protein, p17. . J. Virol, 71, 3474–3483. 9094619 10.1128/jvi.71.5.3474-3483.1997 PMC191494

[23] Lorizate M, Kräusslich HG, (2011). Role of lipids in virus replication. Cold Spring Harb. Perspect. Biol, 3, 1–20, 10.1101/cshperspect.a004820. PMC3179339 21628428

[24] Waheed AA, Freed EO, (2018). The Role of Lipids in Retroviral Replication. Elsevier Inc., 2018 10.1016/B978-0-12-811185-7.00010-8.

[25] Dick RA, Vogt VM, (2014). Membrane interaction of retroviral Gag proteins. Front. Microbiol, 10.3389/fmicb.2014.00187. PMC4010771 24808894

[26] Ono A, Orenstein JM, Freed EO, (2000). Role of the Gag matrix domain in targeting human immunodeficiency virus type 1 assembly. J. Virol, 74, 2855–2866, 10.1128/jvi.74.6.2855-2866.2000. 10684302 PMC111776

[27] Vlach J, Saad JS, (2015). Structural and molecular determinants of HIV-1 Gag binding to the plasma membrane. Front. Microbiol, 10.3389/fmicb.2015.00232. PMC4367181 25852680

[28] Saad JS, Miller J, Tai J, Kim A, Ghanam RH, Summers MF, (2006). Structural basis for targeting HIV-1 Gag proteins to the plasma membrane for virus assembly. Proc. Natl. Acad. Sci, 103, 11364–11369, 10.1073/pnas.0602818103. 16840558 PMC1544092

[29] Vlach J, Eastep GN, Ghanam RH, Watanabe SM, Carter CA, Saad JS, (2018). Structural basis for targeting avian sarcoma virus Gag polyprotein to the plasma membrane for virus assembly. J. Biol. Chem, 10.1074/jbc.RA118.003944. PMC6295720 30309983

[30] Llewellyn GN, Grover JR, Olety B, Ono A, (2013). HIV-1 Gag associates with specific uropod-directed microdomains in a manner dependent on its MA highly basic region. J. Virol, 10.1128/jvi.00040-13. PMC3648103 23536680

[31] Alfadhli A, McNett H, Tsagli S, Bächinger HP, Peyton DH, Barklis E, (2011). HIV-1 matrix protein binding to RNA. J. Mol. Biol, 10.1016/j.jmb.2011.04.063. PMC3139429 21762806

[32] Alfadhli A, Barklis E, (2014). The roles of lipids and nucleic acids in HIV-1 assembly. Front. Microbiol, 5, 1–11, 10.3389/fmicb.2014.00253. 24917853 PMC4042026

[33] Chukkapalli V, Oh SJ, Ono A, (2010). Opposing mechanisms involving RNA and lipids regulate HIV-1 Gag membrane binding through the highly basic region of the matrix domain. Proc. Natl. Acad. Sci, 107, 1600–1605, 10.1073/pnas.0908661107. 20080620 PMC2824378

[34] Brugger B, Glass B, Haberkant P, Leibrecht I, Wieland FT, Kräusslich HG, (2006). The HIV lipidome: a raft with an unusual composition. Proc. Natl. Acad. Sci, 103, 2641–2646, 10.1073/pnas.0511136103. 16481622 PMC1413831

[35] Lorizate M, Sachsenheimer T, Glass B, Habermann A, Gerl MJ, Kräusslich HG, Brügger B, (2013). Comparative lipidomics analysis of HIV-1 particles and their producer cell membrane in different cell lines. Cell. Microbiol, 15, 292–304, 10.1111/cmi.12101. 23279151

[36] Tedbury PR, Freed EO, (2014). The role of matrix in HIV-1 envelope glycoprotein incorporation. Trends Microbiol, 10.1016/j.tim.2014.04.012. PMC4157688 24933691

[37] Checkley MA, Luttge BG, Freed EO, (2011). HIV-1 envelope glycoprotein biosynthesis, trafficking, and incorporation. J. Mol. Biol, 10.1016/j.jmb.2011.04.042. PMC3139147 21762802

[38] Wyma DJ, Jiang J, Shi J, Zhou J, Lineberger JE, Miller MD, Aiken C, (2004). Coupling of human immunodeficiency virus type 1 fusion to virion maturation: a novel role of the gp41 cytoplasmic tail. J. Virol, 10.1128/jvi.78.7.3429-3435.2004. PMC371074 15016865

[39] Jiang J, Aiken C, (2007). Maturation-dependent human immunodeficiency virus type 1 particle fusion requires a carboxyl-terminal region of the gp41 cytoplasmic tail. J. Virol, 10.1128/jvi.00592-07. PMC2045384 17609279

[40] Cosson P, (1996). Direct interaction between the envelope and matrix proteins of HIV-1. . EMBO J., 15, (21) 5783–5788. 8918455 PMC452325

[41] West JT, Weldon SK, Wyss S, Lin X, Yu Q, Thali M, Hunter E, (2002). Mutation of the dominant endocytosis motif in human immunodeficiency virus type 1 gp41 can complement matrix mutations without increasing Env incorporation. J. Virol, 10.1128/jvi.76.7.3338-3349.2002. PMC136014 11884559

[42] Postler TS, Desrosiers RC, (2012). The tale of the long tail: the cytoplasmic domain of HIV-1 gp41. J. Virol, 87, 2–15, 10.1128/jvi.02053-12. 23077317 PMC3536369

[43] Muranyi W, Malkusch S, Müller B, Heilemann M, Kräusslich HG, (2013). Super-resolution microscopy reveals specific recruitment of HIV-1 envelope proteins to viral assembly sites dependent on the envelope C-terminal tail. PLoS Pathog., 9, e1003198 10.1371/journal.ppat.1003198. 23468635 PMC3585150

[44] Wyma DJ, Kotov A, Aiken C, (2002). Evidence for a stable interaction of gp41 with Pr55Gag in immature human immunodeficiency virus type 1 particles. J. Virol, 10.1128/jvi.74.20.9381-9387.2000. PMC112366 11000206

[45] Freed EO, Martin MA, (1995). Virion incorporation of envelope glycoproteins with long but not short cytoplasmic tails is blocked by specific, single amino acid substitutions in the human immunodeficiency virus type 1 matrix. J. Virol, 10.1128/JVI.69.3.1984-1989.1995. PMC188822 7853546

[46] Davis MR, Jiang J, Zhou J, Freed EO, Aiken C, (2006). A mutation in the human immunodeficiency virus type 1 Gag protein destabilizes the interaction of the envelope protein subunits gp120 and gp41. J. Virol, 10.1128/jvi.80.5.2405-2417.2006. PMC1395406 16474147

[47] Mammano F, Kondo E, Sodroski J, Bukovsky A, Göttlinger HG, (1995). Rescue of human immunodeficiency virus type 1 matrix protein mutants by envelope glycoproteins with short cytoplasmic domains. . J. Virol, 69, (6) 3824–3830. 7745730 10.1128/jvi.69.6.3824-3830.1995 PMC189100

[48] Tedbury PR, Ablan SD, Freed EO, (2013). Global rescue of defects in HIV-1 envelope glycoprotein incorporation: implications for matrix structure. PLoS Pathog., 10.1371/journal.ppat.1003739. PMC3828165 24244165

[49] Murakami T, Freed EO, (2002). Genetic evidence for an interaction between human immunodeficiency virus type 1 matrix and alpha-helix 2 of the gp41 cytoplasmic tail. J. Virol, 10.1128/jvi.74.8.3548-3554.2000. PMC111863 10729129

[50] Tedbury PR, Mercredi PY, Gaines CR, Summers MF, Freed EO, (2015). Elucidating the mechanism by which compensatory mutations rescue an HIV-1 matrix mutant defective for Gag membrane targeting and envelope glycoprotein incorporation. J. Mol. Biol, 427, 1413–1427, 10.1016/j.jmb.2015.01.018. 25659909 PMC4844178

[51] Roy NH, Chan J, Lambele M, Thali M, (2013). Clustering and mobility of HIV-1 Env at viral assembly sites predict its propensity to induce cell–cell fusion. J. Virol, 87, 7516–7525, 10.1128/jvi.00790-13. 23637402 PMC3700267

[52] von Schwedler UK, Stray KM, Garrus JE, Sundquist WI, (2003). Functional surfaces of the human immunodeficiency virus type 1 capsid protein. J. Virol, 10.1128/jvi.77.9.5439-5450.2003. PMC153941 12692245

[53] Ganser-Pornillos BK, von Schwedler UK, Stray KM, Aiken C, Sundquist WI, (2004). Assembly properties of the human immunodeficiency virus type 1 CA protein. J. Virol, 78, 2545–2552, 10.1128/JVI.78.5.2545. 14963157 PMC369201

[54] Gres AT, Kirby KA, KewalRamani VN, Tanner JJ, Pornillos O, Sarafianos SG, (2015). X-ray crystal structures of native HIV-1 capsid protein reveal conformational variability. Science, 349, 99–103, 10.1126/science.aaa5936. 26044298 PMC4584149

[55] Adamson CS, Jones IM, (2004). The molecular basis of HIV capsid assembly—five years of progress. Rev. Med. Virol, 10.1002/rmv.418. 15027003

[56] Gamble TR, Yoo S, Vajdos FF, Von Schwedler UK, Worthylake DK, Wang H, McCutcheon JP, Sundquist WI, , (1997). Structure of the carboxyl-terminal dimerization domain of the HIV-1 capsid protein. Science, 10.1126/science.278.5339.849. 9346481

[57] Ivanov D, V Tsodikov O, Kasanov J, Ellenberger T, Wagner G, Collins T, Domain-swapped dimerization of the HIV-1 capsid C-terminal domain. Proc. Natl. Acad. Sci. U. S. A, 104 (2007). 10.1073/pnas.0609477104 PMC1838606 17360528

[58] Ganser-Pornillos BK, Cheng A, Yeager M, (2007). Structure of full-length HIV-1 CA: a model for the mature capsid lattice. Cell, 131, 70–79, 10.1016/J.CELL.2007.08.018. 17923088

[59] Wagner JM, Zadrozny KK, Chrustowicz J, Purdy MD, Yeager M, Ganser-Pornillos BK, Pornillos O, (2016). Crystal structure of an HIV assembly and maturation switch. . Elife, 5, 1–18. 10.7554/eLife.17063 PMC4946879 27416583

[60] Datta SAK, Clark PK, Fan L, Ma B, Harvin DP, Sowder RC, Nussinov R, Wang Y-X, , (2016). Dimerization of the SP1 region of HIV-1 Gag induces a helical conformation and association into helical bundles: implications for particle assembly. J. Virol, 10.1128/jvi.02061-15. PMC4733982 26637452

[61] Datta SAK, Temeselew LG, Crist RM, Soheilian F, Kamata A, Mirro J, Harvin D, Nagashima K, , (2011). On the role of the SP1 domain in HIV-1 particle assembly: a molecular switch?. J. Virol, 85, 4111–4121, 10.1128/JVI.00006-11. 21325421 PMC3126284

[62] Schur FKM, Obr M, Hagen WJH, Wan W, Jakobi AJ, Kirkpatrick JM, Sachse C, Kräusslich HG, , (2016)An atomic model of HIV-1 capsid-SP1 reveals structures regulating assembly and maturation. . Science, 353, 506–508 10.1126/science.aaf9620. 27417497

[63] Liang C, Hu J, Russell RS, Roldan A, Kleiman L, Wainberg MA, (2002). Characterization of a putative a-helix across the capsid-SP1 boundary that is critical for the multimerization of human immunodeficiency virus type 1 Gag. J. Virol, 76, 10.1128/jvi.76.22.11729-11737.2002. PMC136778 12388733

[64] Guo X, Roldan A, Hu J, Wainberg MA, Liang C, (2005). Mutation of the SP1 sequence impairs both multimerization and membrane-binding activities of human immunodeficiency virus type 1 Gag. J. Virol, 10.1128/jvi.79.3.1803-1812.2005. PMC544129 15650204

[65] Guo X, Liang C, (2005). Opposing effects of the M368A point mutation and deletion of the SP1 region on membrane binding of human immunodeficiency virus type 1 Gag. Virology, 10.1016/j.virol.2005.03.004. 15840522

[66] Tan X, Thapa N, Choi S, Anderson RA, (2015). Emerging roles of PtdIns(4,5)P2 - beyond the plasma membrane. J. Cell Sci, 128, 4047–4056, 10.1242/jcs.175208. 26574506 PMC4712784

[67] Wen Y, Vogt VM, Feigenson GW, (2018). Multivalent cation-bridged PI(4,5)P2 clusters form at very low concentrations. Biophys. J, 114, 2630–2639, 10.1016/j.bpj.2018.04.048. 29874613 PMC6129474

[68] Laux T, Fukami K, Thelen M, Golub T, Frey D, Caroni P, (2000). Gap43, Marcks, and Cap23 modulate Pi(4,5)p_2_ at plasmalemmal rafts, and regulate cell cortex actin dynamics through a common mechanism. J. Cell Biol, 149, 1455–1472, 10.1083/jcb.149.7.1455. 10871285 PMC2175130

[69] Bethoney KA, King MC, Lemmon MA, Ostap EM, Hinshaw JE, (2009). A possible effector role for the pleckstrin homology (PH) domain of dynamin. Proc. Natl. Acad. Sci, 106, 13359–13364, 10.1073/pnas.0906945106. 19666604 PMC2720410

[70] Kwiatkowska K, (2010). One lipid, multiple functions: How various pools of PI(4,5)P2 are created in the plasma membrane. Cell. Mol. Life Sci, 67, 3927–3946, 10.1007/s00018-010-0432-5. 20559679 PMC11115911

[71] Shaner NC, Lambert GG, Chammas A, Ni Y, Cranfill PJ, Baird MA, Sell BR, Allen JR, , (2013). A bright monomeric green fluorescent protein derived from Branchiostoma lanceolatum. Nat. Methods, 10.1038/nmeth.2413. PMC3811051 23524392

[72] Ferguson KM, Lemmon MA, Schlessinger J, Sigler PB, (1995). Structure of the high affinity complex of inositol trisphosphate with a phospholipase C pleckstrin homology domain. Cell, 83, 1037–1046, 10.1016/0092-8674(95)90219-8. 8521504

[73] Szentpetery Z, Balla A, Kim YJ, Lemmon MA, Balla T, (2009). Live cell imaging with protein domains capable of recognizing phosphatidylinositol 4,5-bisphosphate; a comparative study. BMC Cell Biol., 10, 67, 10.1186/1471-2121-10-67. 19769794 PMC2755470

[74] Bilkova E, Pleskot R, Rissanen S, Sun S, Czogalla A, Cwiklik L, Róg T, Vattulainen I, , (2017). Calcium directly regulates phosphatidylinositol 4,5-bisphosphate headgroup conformation and recognition. J. Am. Chem. Soc, 139, 4019–4024, 10.1021/jacs.6b11760. 28177616 PMC5364432

[75] Wang J, Arbuzova A, Hangyas-Mihalyne G, McLaughlin S, (2001). The effector domain of myristoylated alanine-rich C kinase substrate binds strongly to phosphatidylinositol 4,5-bisphosphate. J. Biol. Chem, 276, 5012–5019, 10.1074/jbc.M008355200. 11053422

[76] Wang J, Gambhir A, Hangyás-Mihályné G, Murray D, Golebiewska U, McLaughlin S, (2002). Lateral sequestration of phosphatidylinositol 4,5-bisphosphate by the basic effector domain of myristoylated alanine-rich C kinase substrate is due to nonspecific electrostatic interactions. J. Biol. Chem, 277, 34401–34412, 10.1074/jbc.M203954200. 12097325

[77] Denisov G, Wanaski S, Luan P, Glaser M, McLaughlin S, (1998). Binding of basic peptides to membranes produces lateral domains enriched in the acidic lipids phosphatidylserine and phosphatidylinositol 4,5-bisphosphate: An electrostatic model and experimental results. Biophys. J, 74, 731–744, 10.1016/S0006-3495(98)73998-0. 9533686 PMC1302554

[78] Dick RA, Kamynina E, Vogt VM, (2013). Effect of multimerization on membrane association of Rous sarcoma virus and HIV-1 matrix domain proteins. J. Virol, 87, 13598–13608, 10.1128/jvi.01659-13. 24109216 PMC3838224

[79] McLaughlin S, Wang J, Gambhir A, Murray D, (2002). PIP_2_ and proteins: interactions, organization, and information flow. Annu. Rev. Biophys. Biomol. Struct, 31, 151–175, 10.1146/annurev.biophys.31.082901.134259. 11988466

[80] Zaitseva I, Cafiso DS, Wang J, Murray D, Gambhir A, Pentyala SN, Smith SO, Mclaughlin S, , (2004). Electrostatic sequestration of PIP2 on phospholipid membranes by basic/aromatic regions of proteins. Biophys. J, 86, 2188–2207, 10.1016/S0006-3495(04)74278-2. 15041659 PMC1304070

[81] Rauch ME, Ferguson CG, Prestwich GD, Cafiso DS, (2002). Myristoylated alanine-rich C kinase substrate (MARCKS) sequesters spin-labeled phosphatidylinositol 4,5-bisphosphate in lipid bilayers. J. Biol. Chem, 277, 14068–14076, 10.1074/jbc.M109572200. 11825894

[82] Schur FKM, Dick RA, Hagen WJH, Vogt VM, Briggs JAG, (2015). The structure of immature virus-like rous sarcoma virus Gag particles reveals a structural role for the p10 domain in assembly. J. Virol, 89, 10294–10302, 10.1128/JVI.01502-15. 26223638 PMC4580193

[83] Dick RA, Barros M, Jin D, Lösche M, Vogt VM, (2016). Membrane binding of the rous sarcoma virus Gag protein is cooperative and dependent on the spacer peptide assembly domain. J. Virol, 90, 2473–2485, 10.1128/JVI.02733-15. PMC4810714 26676779

[84] Kerfeld CA, Kerfeld CA, Sawaya MR, Tanaka S, Nguyen CV, Phillips M, Beeby M, Yeates TO, (2005). Protein structures forming the shell of primitive bacterial organelles. Science, 10.1126/science.1113397. 16081736

[85] Pornillos O, Ganser-Pornillos BK, Kelly BN, Hua Y, Whitby FG, Stout CD, Sundquist WI, Hill CP, , (2009). X-ray structures of the hexameric building block of the HIV capsid. Cell, 137, 1282–1292, 10.1016/j.cell.2009.04.063. 19523676 PMC2840706

[86] Dalton AK, Ako-adjei D, Murray PS, Murray D, Vogt M, (2007). Electrostatic interactions drive membrane association of the human immunodeficiency virus type 1 Gag MA domain. J. Virol, 81, 6434–6445, 10.1128/JVI.02757-06. 17392361 PMC1900125

[87] Tang C, Loeliger E, Luncsford P, Kinde I, Beckett D, Summers MF, (2004). Entropic switch regulates myristate exposure in the HIV-1 matrix protein. Proc. Natl. Acad. Sci. U. S. A, 101, 517–522, 10.1073/pnas.0305665101. 14699046 PMC327179

[88] Alfadhli A, Barklis RL, Barklis E, (2009). HIV-1 matrix organizes as a hexamer of trimers on membranes containing phosphatidylinositol-(4,5)-bisphosphate. Virology, 387, 466–472, 10.1016/j.virol.2009.02.048. 19327811 PMC2680355

[89] Ono A, Freed EO, (2004). Cell-type-dependent targeting of human immunodeficiency virus type 1 assembly to the plasma membrane and the multivesicular body. J. Virol, 10.1128/jvi.78.3.1552-1563.2004. PMC321403 14722309

[90] Hill CP, Worthylake D, Bancroft DP, Christensen AM, Sundquist WI, (1996). Crystal structures of the trimeric human immunodeficiency virus type 1 matrix protein: implications for membrane association and assembly. . Proc. Natl. Acad. Sci. U. S. A, 93, 3099–3104 http://www.ncbi.nlm.nih.gov/pubmed/8610175 (accessed January 5, 2019). 8610175 10.1073/pnas.93.7.3099 PMC39768

[91] Joshi A, Ablan SD, Soheilian F, Nagashima K, Freed EO, (2009). Evidence that productive human immunodeficiency virus type 1 assembly can occur in an intracellular compartment. J. Virol, 83, 5375–5387, 10.1128/JVI.00109-09. 19297499 PMC2681934

[92] Massiah MA, Worthylake D, Christensen AM, Sundquist WI, Hill CP, Summers MF, (1996). Comparison of the NMR and X-ray structures of the HIV-1 matrix protein: evidence for conformational changes during viral assembly. Protein Sci., 5, 2391–2398, 10.1002/pro.5560051202. 8976548 PMC2143307

[93] Massiah MA, Starich MR, Paschall C, Summers MF, Christensen AM, Sundquist WI, (1994). Three-dimensional structure of the human immunodeficiency virus type 1 matrix protein. J. Mol. Biol, 10.1006/jmbi.1994.1719. 7966331

[94] Alfadhli A, Huseby D, Kapit E, Colman D, Barklis E, (2006). Human immunodeficiency virus type 1 matrix protein assembles on membranes as a hexamer. J. Virol, 81, 1472–1478, 10.1128/jvi.02122-06. 17108052 PMC1797500

[95] Tedbury PR, Novikova M, Ablan SD, Freed EO, (2016). Biochemical evidence of a role for matrix trimerization in HIV-1 envelope glycoprotein incorporation. Proc. Natl. Acad. Sci. U. S. A, 113, E182–E190, 10.1073/pnas.1516618113. 26711999 PMC4720328

[96] Alfadhli A, Mack A, Ritchie C, Cylinder I, Harper L, Tedbury PR, Freed EO, Barklis E, (2016). Trimer enhancement mutation effects on HIV-1 matrix protein binding activities. J. Virol, 90, 5657–5664, 10.1128/JVI.00509-16. 27030269 PMC4886772

[97] Datta SAK, Zhao Z, Clark PK, Tarasov S, Alexandratos JN, Campbell SJ, Kvaratskhelia M, Lebowitz J, , (2007). Interactions between HIV-1 Gag molecules in solution: an inositol phosphate-mediated switch. J. Mol. Biol, 10.1016/j.jmb.2006.10.072. PMC1829305 17098251

[98] Datta SAK, Curtis JE, Ratcliff W, Clark PK, Crist RM, Lebowitz J, Krueger S, Rein A, (2007). Conformation of the HIV-1 Gag protein in solution. J. Mol. Biol, 10.1016/j.jmb.2006.10.073. PMC1866279 17097677

[99] Yandrapalli N, Lubart Q, Tanwar HS, Picart C, Mak J, Muriaux D, Favard C, (2016). Self assembly of HIV-1 Gag protein on lipid membranes generates PI(4,5)P2/cholesterol nanoclusters. Sci. Rep, 6, 10.1038/srep39332. PMC5180241 28008947

[100] Dick RA, Xu C, Morado DR, Kravchuk V, Ricana CL, Lyddon TD, Broad AM, Feathers JR, , (2020). Structures of immature EIAV Gag lattices reveal a conserved role for IP6 in lentivirus assembly. PLoS Pathog., 16, e1008277 10.1371/journal.ppat.1008277. 31986188 PMC7004409

[101] Qu K, Glass B, Doležal M, Schur FKM, Murciano B, Rein A, Rumlová M, Ruml TT, , (2018). Structure and architecture of immature and mature murine leukemia virus capsids. Proc. Natl. Acad. Sci. U. S. A, 115, E11751–E11760 10.1073/pnas.1811580115. 30478053 PMC6294937

[102] Mattei S, Tan A, Glass B, Müller B, Kräusslich HG, Briggs JAG, (2018). High-resolution structures of HIV-1 Gag cleavage mutants determine structural switch for virus maturation. . Proc. Natl. Acad. Sci. U. S. A, 115, E9401–E9410 http://www.ncbi.nlm.nih.gov/pubmed/30217893 (accessed May 5, 2020). 30217893 10.1073/pnas.1811237115 PMC6176557

[103] Mattei S, Glass B, Hagen WJH, Kräusslich HG, Briggs JAG, (2016). The structure and flexibility of conical HIV-1 capsids determined within intact virions. Science, 354, 1434–1437, 10.1126/science.aah4972. 27980210

[104] Schur FKM, Hagen WJH, Rumlová M, Ruml T, Müller B, Kraüsslich HG, Briggs JAG, (2015). Structure of the immature HIV-1 capsid in intact virus particles at 8.8 Å resolution. Nature, 517, 505–508, 10.1038/nature13838. 25363765

[105] Accola MA, Höglund S, Göttlinger HG, Sundquist WI, (1998). A putative alpha-helical structure which overlaps the capsid-p2 boundary in the human immunodeficiency virus type 1 Gag precursor is crucial for viral particle assembly. J. Virol, 72, 2072–2078, 10.1128/jvi.77.9.5439-5450.2003. 9499062 PMC109501

[106] Gross I, Hohenberg H, Wilk T, Wiegers K, Grättinger M, Müller B, Fuller S, Kräusslich HG, (2000). A conformational switch controlling HIV-1 morphogenesis. EMBO J., 19, 103–113, 10.1093/emboj/19.1.103. 10619849 PMC1171782

[107] Wen Y, Dick RA, Feigenson GW, Vogt VM, (2016). Effects of membrane charge and order on membrane binding of the retroviral structural protein Gag. J. Virol, 10.1128/jvi.01102-16. PMC5044813 27512076

[108] Romani A, (2007). Regulation of magnesium homeostasis and transport in mammalian cells. Arch. Biochem. Biophys, 458, 90–102, 10.1016/j.abb.2006.07.012. 16949548

[109] Lemmon MA, Ferguson KM, (2000). Signal-dependent membrane targeting by pleckstrin homology (PH) domains. . Biochem. J, 350, (Pt 1) 1–18. 10926821 PMC1221219

[110] Malakhov MP, Mattern MR, Malakhova OA, Drinker M, Weeks SD, Butt TR, (2004). SUMO fusions and SUMO-specific protease for efficient expression and purification of proteins. J. Struct. Funct. Genom, 5, 75–86, 10.1023/B:JSFG.0000029237.70316.52. 15263846

[111] Kingsley PB, Feigenson GW, (1979). The synthesis of a perdeuterated phospholipid: 1,2-dimyristoyl-sn-glycero-3-phosphocholine-d72. Chem. Phys. Lipids, 10.1016/0009-3084(79)90083-5.

[112] Wang T, (2010). Inductively coupled plasma optical emission spectrometry. Anal. Instrum. Handbook, Second ed., 2010 10.1201/9780849390395.ch3.

[113] Buboltz JT, Feigenson GW, (1999). A novel strategy for the preparation of liposomes: rapid solvent exchange. Biochim. Biophys. Acta Biomembr, 1417, 232–245, 10.1016/S0005-2736(99)00006-1. 10082799

[114] Mastronarde DN, (2005). Automated electron microscope tomography using robust prediction of specimen movements. J. Struct. Biol, 152, 36–51, 10.1016/j.jsb.2005.07.007. 16182563

[115] Hagen WJH, Wan W, Briggs JAG, (2017). Implementation of a cryo-electron tomography tilt-scheme optimized for high resolution subtomogram averaging. J. Struct. Biol, 197, 191–198, 10.1016/j.jsb.2016.06.007. 27313000 PMC5287356

[116] Kremer JR, Mastronarde DN, McIntosh JR, (1996). Computer visualization of three-dimensional image data using IMOD. J. Struct. Biol, 116, 71–76, 10.1006/jsbi.1996.0013. 8742726

