# Supplementary material for: Mechanisms of PI(4,5)P2 Enrichment in HIV-1 Viral Membranes
Source: J Mol Biol. Author manuscript; Available in PMC 2021 Jul 7. (PMC8262684; doi:10.1016/j.jmb.2020.07.018)
Supplement: Supplementary data [file NIHMS1717033-supplement-Supplementary_data.pdf]

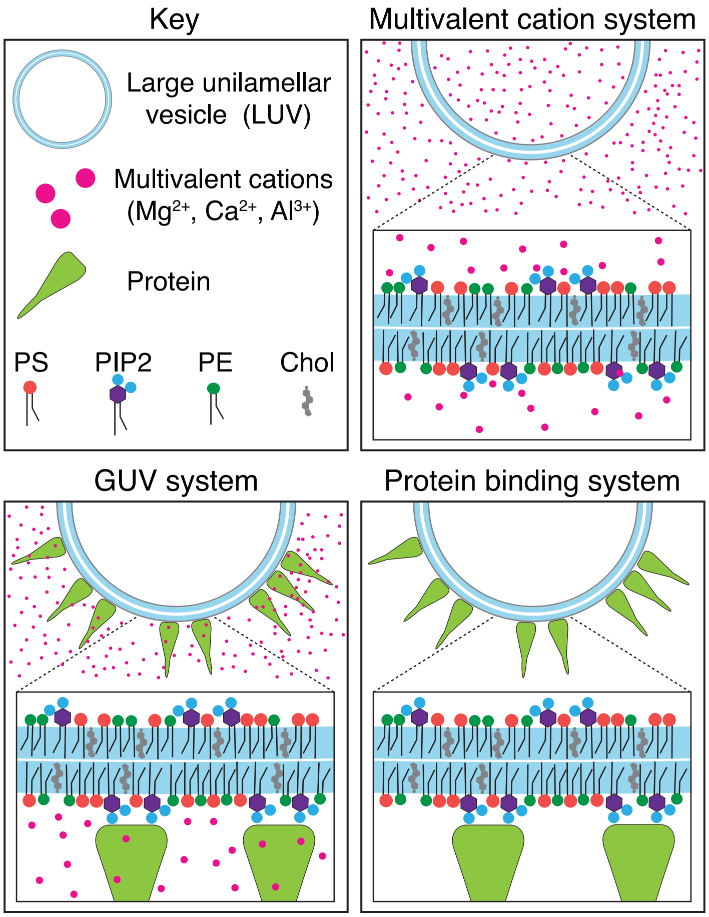

Figure S1. Illustration of fluorescence quenching and membrane binding experiments. All lipid bilayers were prepared with PE/PS/Chol/PIP2 (32/30/2/36 mol%). In the GUV system, mNG-tagged protein (green) and metal ions (pink) are added only to the outside of the GUVs. In the multivalent cation system, the liposomes were prepared in the presence of the ions, so that both leaflets of the bilayer are exposed equally. In this case PIP2 in both leaflets is affected equally, resulting in a maximum effect from multivalent cations. In the protein binding system, protein was added after liposome preparation, so that the protein acts only on the outer leaflet of the bilayer, and thus only one half of the PIP2 is affected.

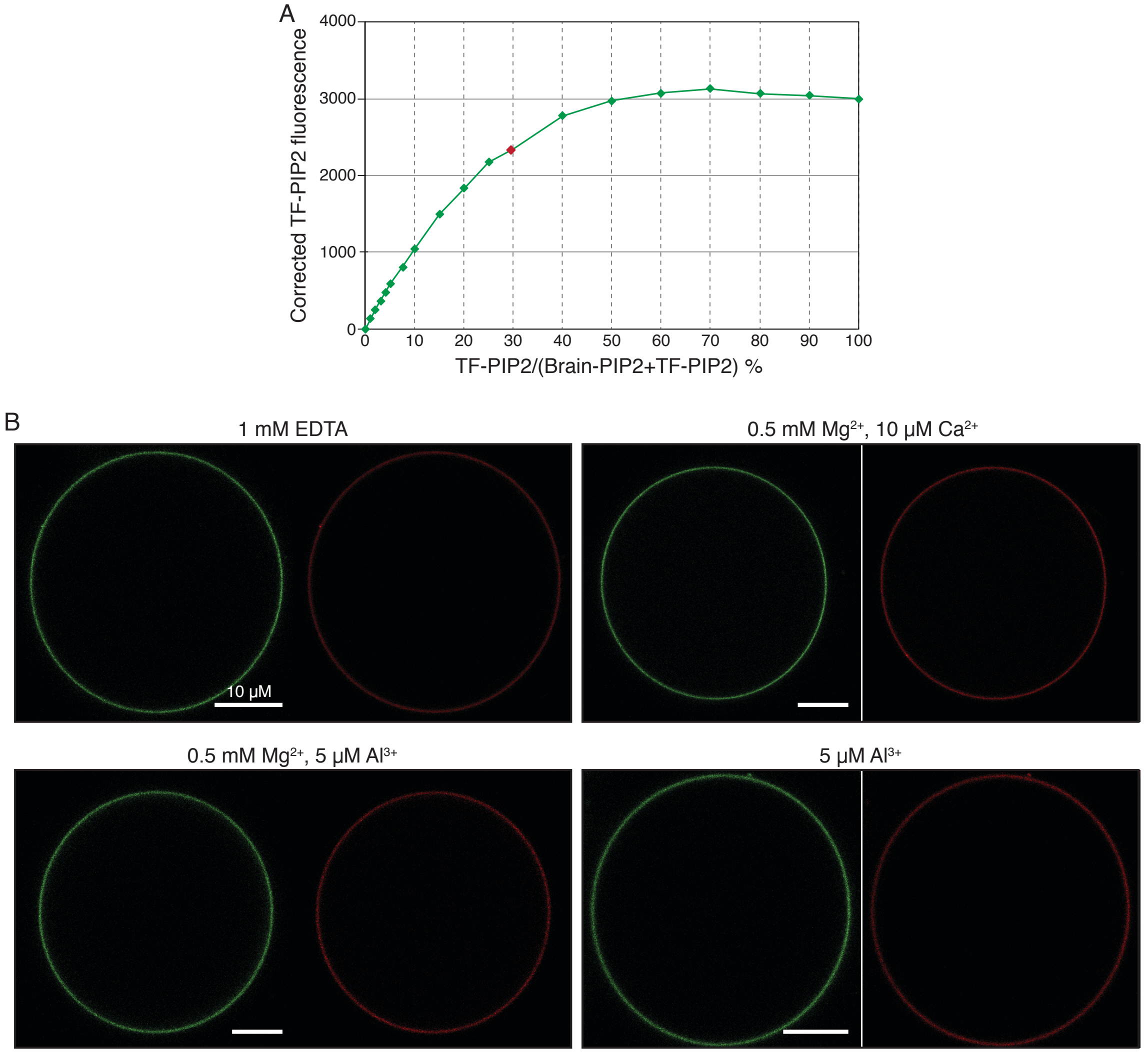

Figure S2. Different ratios of Brain-PIP2/TF-PIP2 provide different assay sensitivity. (A) In LUVs composed of POPE/POPS/Chol/PIP2 (32/30/36/2), the 2 mol % total PIP2 was a mixture of Brain-PIP2 and TF-PIP2 at different ratios. The fraction of TF-PIP2 of the total PIP2 is shown on the X-axis. All LUVs were prepared in 100mM KCl, 20mM HEPES, pH = 7.2, with 0.5 mM Mg^{2+} and 10 μM Ca^{2+} to induce PIP2 clustering. TF-PIP2 fluorescence was measured from 500 μM LUVs. The optimal ratio of Brain PIP2/TF-PIP2 was chosen at 7/3 = 30 % TF-PIP2 for all experiments reported here, indicated by the red dot. (B) Confocal microscope image of GUVs [POPE/POPS/Chol/brain-PIP2 (32/30/36/2)] prepared with 90/5/5 % brain-PIP2 to TF-PIP2 (red) and TMR-PIP2 (green). All scale bars are 10 μm.

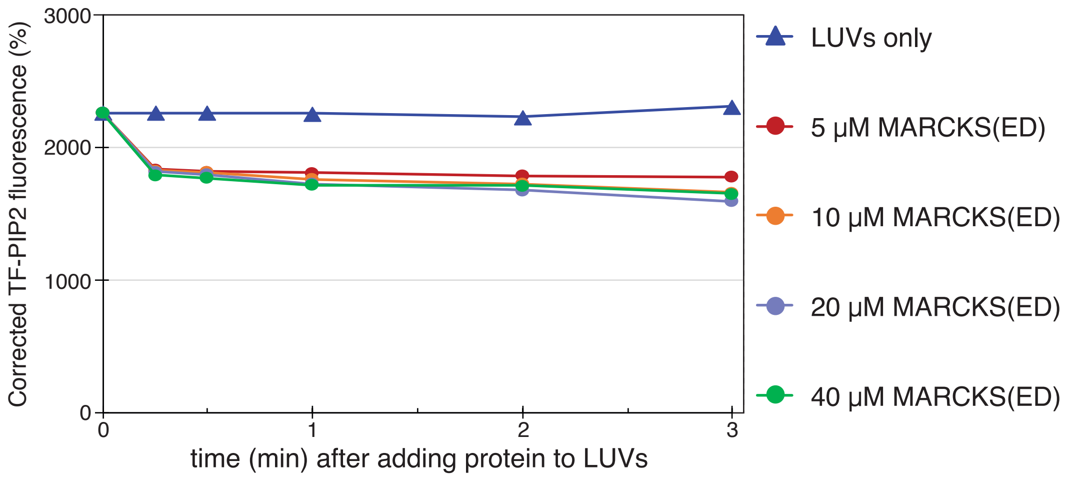

Figure S3. Concentration dependence of PIP2 clustering induced by MARCKS(ED) peptide. PIP2-containing LUVs were prepared in EDTA as in Figure 2. A total volume of 40 μl MARCKS(ED) peptide was added to the outside of 160 μl LUVs and thoroughly mixed to reach a final concentration of 5, 10, 20, or 40 μM. TF-PIP2 fluorescence was measured at 0.25, 0.5, 1, 2, and 3 min post-mixing. Over this concentration range, the peptide was equally effective at inducing self-quenching of TF-PIP2

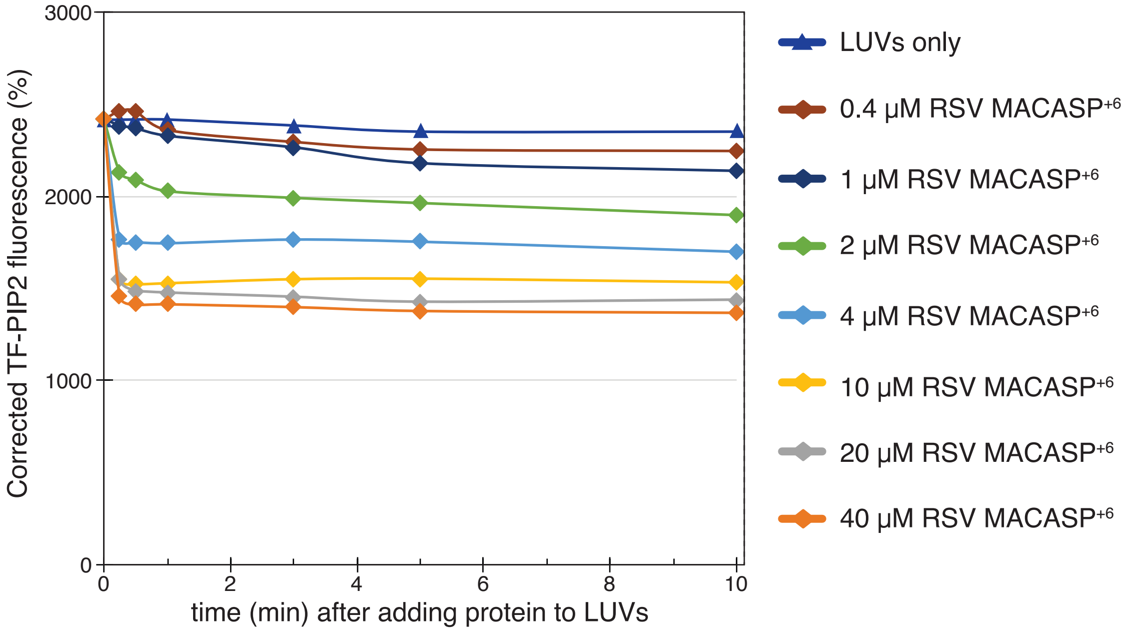

Figure S4. Concentration dependence of PIP2 clustering induced by RSV MACASP^{+6}. LUVs in EDTA were prepared as in Figure 2. A total volume of 40 µl RSV MASP^{+6} and buffer was added to the outside of 160 µl LUVs at 500 µM and thoroughly mixed, to yield protein concentrations from 0.4 µM to 40 µM. TF-PIP2 fluorescence was measured at 0.25, 0.5, 1, 2, and 3 min post-mixing. Maximum self-quenching was induced by protein at 20 µM.

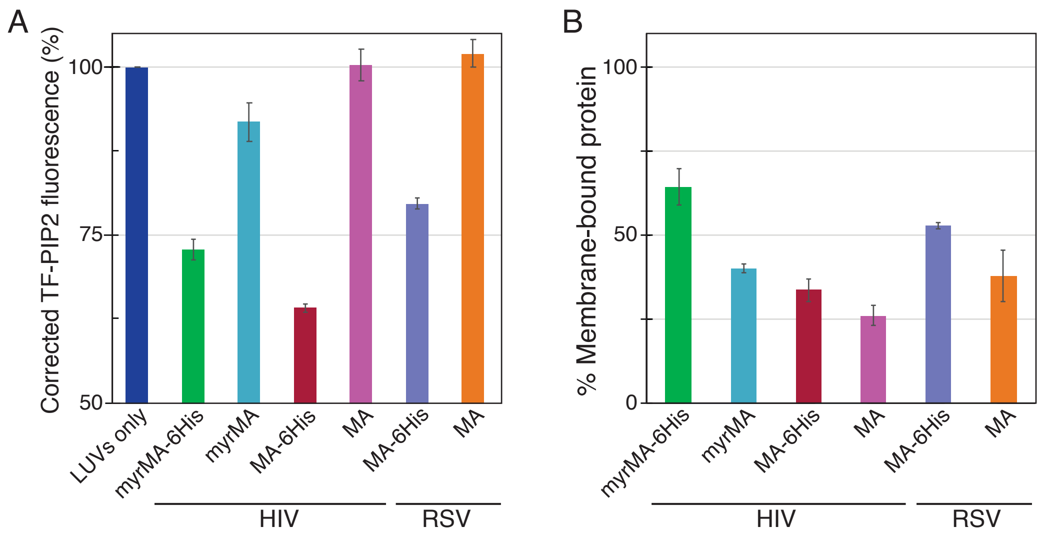

Figure S5. The C-terminal 6-His tag induces PIP2 clustering and enhances protein membrane binding. PIP2-containing LUVs were prepared in the presence of 1 mM EDTA as described in Figure 2, and a TF-PIP2 self-quenching assay was performed as in Figure 5. (A) Each bar in the graph represents the average TF-PIP2 fluorescence % over the 1, 2, and 3 min time points post-mixing. Each quenching assay was performed at least three times; error bars show the standard deviation from the mean. (B) Membrane binding was determined by pelleting assays for each protein. Each pelleting assay was performed at least three times; error bars show the standard deviation from the mean.

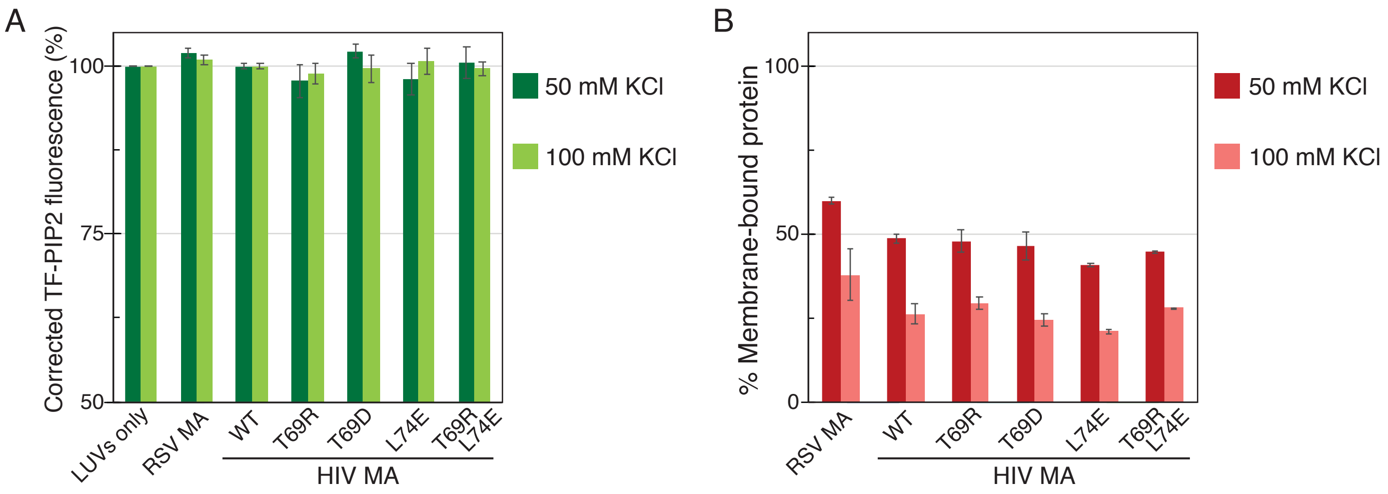

Figure S6. RSV and HIV-1 MA do not promote PIP2 clustering even at lower ionic strength. (A) Effect of ionic strength on wild type RSV and wild type and mutant HIV MA membrane binding on PIP2 clustering. (B) Amount of protein associated with membranes determined by liposome pelleting assays.

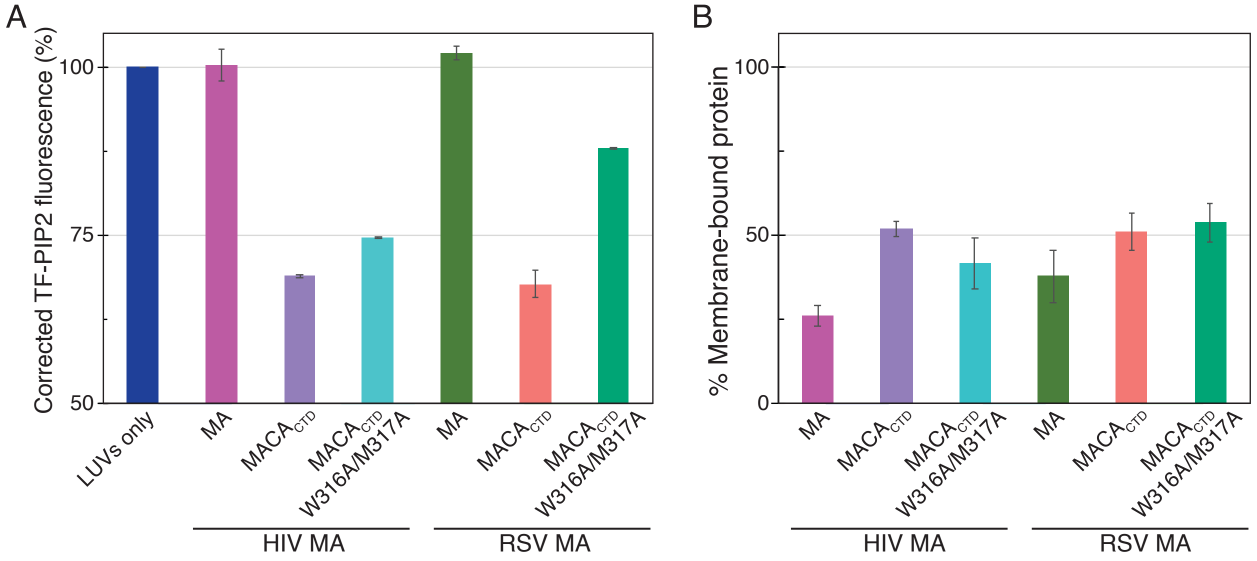

Figure S7. HIV-1 and RSV MA fused with HIV-1 CA_{CTD} WT or WM mutant can induce PIP2 clustering. PIP2-containing LUVs were prepared in the presence of 1mM EDTA as described in Figure 2, and a TF-PIP2 self-quenching assay was performed as in Figure 5. (A) Each bar in the graph represents the average TF-PIP2 fluorescence % over the 1, 2, and 3 min time points post-mixing. Each quenching assay was performed at least three times with error bars showing the standard deviations from the means. (B) Membrane binding of proteins. Each assay was performed at least three times, with error bars showing standard deviations from the means.

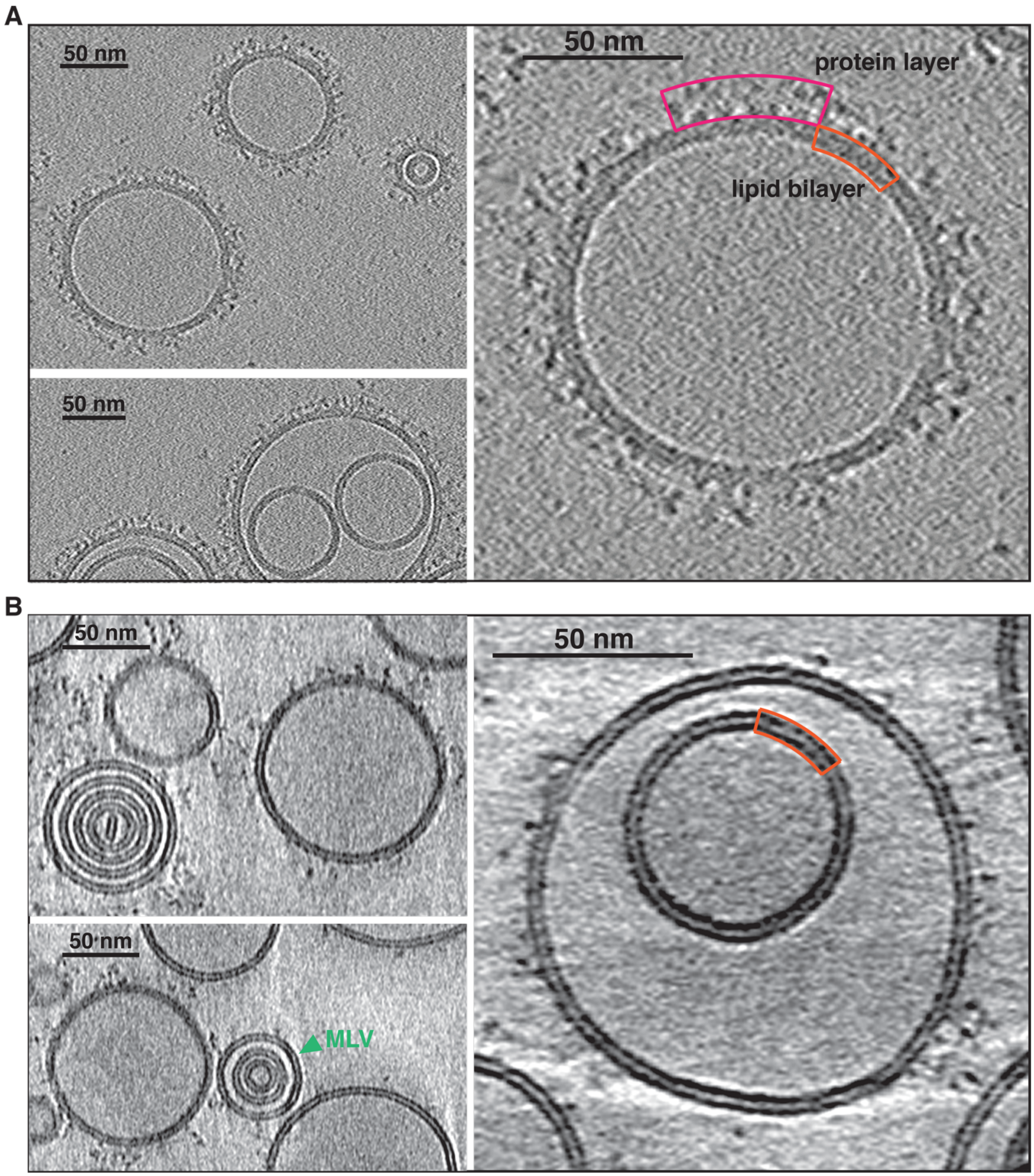

Figure S8. Cryo-electron microscopy images of HIV-1 MACASP bound to LUVs. PIP2-containing LUVs were prepared in the presence of 1mM EDTA as described in Figure 2, and HIV-1 MACASP protein was added as in Figure 8. Approximately 40% of protein was bound to liposomes as determined by pelleting. All images are at bin-factor 8, and are of six summed frames with a z-thickness of 6 nm. (A) Images taken at a defocus of -2.5 μm. Top panel shows protein bound to three liposomes of different sizes. As expected, the protein is bound only on the outer leaflet of the liposomes. The bottom panel shows an example of a liposome with two smaller liposomes inside. The inner liposomes serve as examples of the appearance of membranes without protein bound. Right panel is a blow-up of the large liposome in A. The approximate location of the lipid bilayer (orange) and protein layer (pink) is indicated. (B) Same sample as in A, but images were taken at a defocus of -5 μm. Less protein was observed bound to this field of liposomes, but the higher defocus value results in increased contrast of the lipid headgroups, so that the two leaflets of the bilayer are more clearly visible. Left panels each have an example of a multi-layered liposome. Right panel is a zoomed-in image of a liposome with low amounts of protein bound.

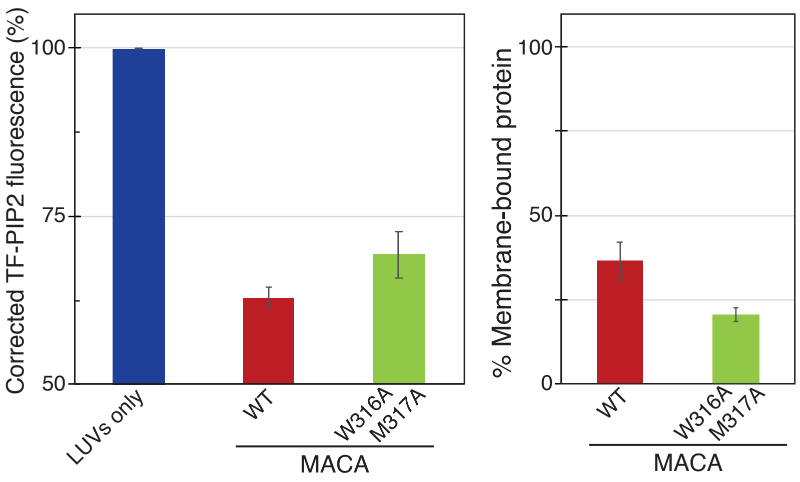

Figure S9. CA_{CTD} dimer mutations in MACA do not alter quenching**.** Effect of W316A/M317A mutation on PIP2 quenching (left) and membrane binding (right) on MACA protein.
